# Evaluation of a Hybrid Neural–Polynomial Deep Q-Network for Switching-Aware Spectrum Selection in a Controlled Radio-Frequency Measurement-Replay Testbed

**DOI:** 10.3390/s26175501

**Published:** 2026-08-30

**Authors:** Yuxuan Pan, Ying Yan, Dingyi Sun, Zhenyu Li, Zhixuan Zhang, Jun Cai, Dapeng Chen, Qi Wu, Zongyuan Shen

**Affiliations:** 1 Reading Academy, Nanjing University of Information Science and Technology, Nanjing 210044, China; ryan_penn@connect.hku.hk; 2 School of Automation, Nanjing University of Information Science and Technology, Nanjing 210044, China; j.cai@nuist.edu.cn (J.C.); dpchen@nuist.edu.cn (D.C.); 3 School of Artificial Intelligence, Nanjing University of Information Science and Technology, Nanjing 210044, China; 202483460018@nuist.edu.cn; 4 Nanjing Research Institute of Electronics Technology, Nanjing 210039, China; zhenyuli_2012@sina.com; 5 School of Electronics & Information Engineering, Nanjing University of Information Science and Technology, Nanjing 210044, China; 20211118009@nuist.edu.cn; 6 Department of Automation, Shanghai Jiao Tong University, Shanghai 200240, China; edmondqwu@sjtu.edu.cn; 7 College of Information Science and Technology, Jinan University, Guangzhou 510632, China; shenzongyuan@jnu.edu.cn

**Keywords:** HNP-DQN, cognitive radar, adaptive spectrum selection, deep reinforcement learning, polynomial feature expansion, channel switching cost

## Abstract

Switching-aware spectrum selection requires balancing interference avoidance against retuning costs. This paper evaluates a hybrid neural–polynomial deep Q-network (HNP-DQN) in an eight-channel, measurement-driven 5 GHz testbed with exogenous sweeping and random interference. The architecture combines learned latent features with an element-wise second-order expansion, layer normalization (LayerNorm), and a dueling Double Deep Q-Network (DDQN) backbone. Files are separated before window construction, and the evaluation includes a pre-inspected pilot and two outcome-uninspected distance and power transfers. The comparison includes strong deterministic rules and a capacity-matched multilayer perceptron (MLP) trained with the same DDQN procedure. All methods are evaluated on common trajectories with paired inference across 10 independently trained seeds and Holm correction. Under deterministic sweeping, the schedule-aware rule was given the declared initial phase and one-channel-per-step direction and used an internal step counter to track the deterministic progression. These schedule variables and the corresponding step index are absent from HNP-DQN’s 48-dimensional observation. The rule matched the trajectory-wise dynamic-programming upper bound and outperformed HNP-DQN in all three settings (differences calculated as HNP-DQN minus the rule: −1.91, −3.51, and −1.97; all adjusted p=0.023). This information-asymmetric operational comparison shows the advantage attainable when the declared sweep specification and its progression are directly exploited; however, it does not provide a matched-information architectural ranking. By contrast, under random jamming, all comparisons between HNP-DQN and the threshold rule were inconclusive after correction, as were all six capacity-matched comparisons between HNP-DQN and the MLP. Of the 24 exploratory ablation comparisons, one favored the γ=0 variant in the random pilot, whereas the other 23 were inconclusive. Accordingly, this paper provides an information-aware, approximately parameter-matched reference for evaluating when explicit knowledge, observation-based control, or additional learning complexity is justified within the declared measurement-replay scope.

## 1. Introduction

The proliferation of wireless communication systems [1] and the increasing density of both military and civilian emitters have transformed modern electromagnetic environments into highly heterogeneous, contested spaces [2,3]. Under these conditions, radar systems must exhibit high levels of real-time adaptability and autonomy to maintain detection and tracking performance. Traditional electronic-protection (EP) measures such as frequency hopping, spread-spectrum transmission, and fixed adaptive filters remain useful against simple or stationary interference, but their performance may degrade under nonstationary and time-varying interference patterns [4]. These trends motivate active, closed-loop anti-jamming paradigms in which the radar senses the spectrum, learns interference patterns, and adapts transmission parameters to maximize long-term mission utility. The cognitive radar concept (sense–learn–adapt) [5] provides a natural theoretical foundation for this perspective. Within this paradigm, reinforcement learning (RL) is one possible tool for adaptive spectrum decision making, but its use must be justified against contextual and deterministic alternatives. In the present problem, the radio-frequency (RF) environment evolves exogenously: channel selection does not change the next measurement window or jammer process. Its limited temporal dependence arises from channel continuity, because the action selected at step *t* determines whether a switch is incurred at step t+1. This motivates a switching-aware sequential formulation while also making strong myopic and deterministic rules essential comparators. The neural–polynomial architecture is evaluated as a structured function approximator; any claimed benefit must be separated from parameter capacity by a capacity-matched conventional multilayer perceptron (MLP).

### 1.1. Cognitive Electronic Warfare and Anti-Jamming

**Figure 1:**
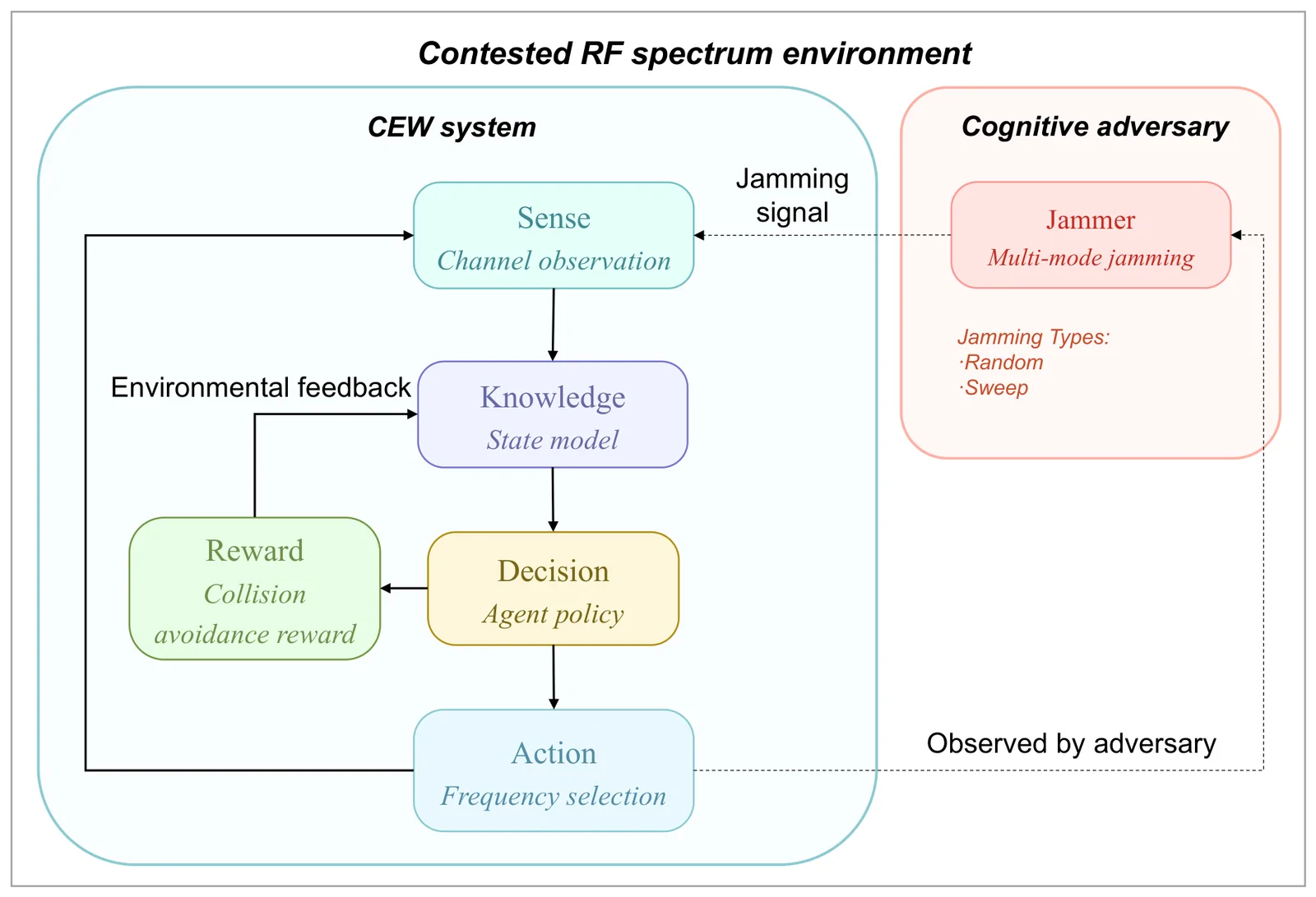
Conceptual cognitive electronic-warfare closed-loop architecture, shown only as broader motivation. The dashed adversary observation/response path is not implemented in the non-reactive measurement-replay evaluation used in this paper.

Cognitive electronic warfare (CEW) extends the cognitive radar paradigm into the broader electronic-warfare domain by endowing sensing systems with perception–learning–adaptation loops that actively shape the electromagnetic battlespace to achieve mission objectives [6]. The cognitive radar concept was originally formalized by Haykin, who framed sensing as a closed-loop process in which the radar continuously senses the environment, updates an internal representation (memory/knowledge), and adapts transmit/receive parameters (waveform, frequency, beam, dwell time) to improve performance under uncertainty. CEW transposes these principles to contested environments where adversaries are intelligent and adaptive, requiring the defender to reason about long-term trade-offs and to anticipate opponents’ strategies. This broader CEW setting should be distinguished from the evaluation in this paper, which considers only non-reactive random and periodic sweeping interference and does not model a jammer that observes, predicts, or learns from the agent. Figure 1 summarizes the broader CEW feedback loop; its dashed adversary-response path is contextual only and is not implemented in the present measurement-replay evaluation.

The importance of CEW and adaptive anti-jamming for modern radar arises from three converging trends. First, the electromagnetic environment is becoming increasingly congested and heterogeneous with the growing coexistence of military, commercial, and civilian emitters that create dense, nonstationary interference patterns [7]. Second, adversaries are deploying intelligent jammers and cognitive electronic-warfare platforms that observe, learn, and adapt their interference tactics in response to the radar’s behavior, thereby challenging static or predesigned countermeasures [8,9]. Third, spectrum scarcity and operational constraints (shared bands, regulatory limitations, and mission-performance requirements) force radars to trade off spectral efficiency, detection performance, and survivability in real time [10]. Together, these factors motivate active, learning-based anti-jamming paradigms that can operate under tight latency, partial observability, and resource constraints [11]. Sequential channel selection provides an abstract decision model relevant to spectrum-agile radar research. However, the 5 GHz environment used here supports only a controlled spectrum-decision-layer testbed evaluation; it does not support radar validation. Claims about radar detection, tracking, radar-band operation, or hardware-level anti-jamming would require separate experiments. Traditional EP techniques such as frequency hopping [12], spread-spectrum transmission [13], and rule-based waveform selection remain valuable; their effectiveness depends on threat model, sensing, and operating assumptions. Frequency-agile schemes can improve survivability, but frequent switching can reduce net mission utility through reconfiguration and spectral costs. Observation-driven decision methods are therefore worth evaluating when policies must adapt to measured context without presuming that they dominate classical or model-based approaches. These trade-offs make baseline strength, model capacity, switching overhead, and out-of-sample validation central design considerations for this paper.

### 1.2. Reinforcement Learning and Function Approximation

**Figure 2:**
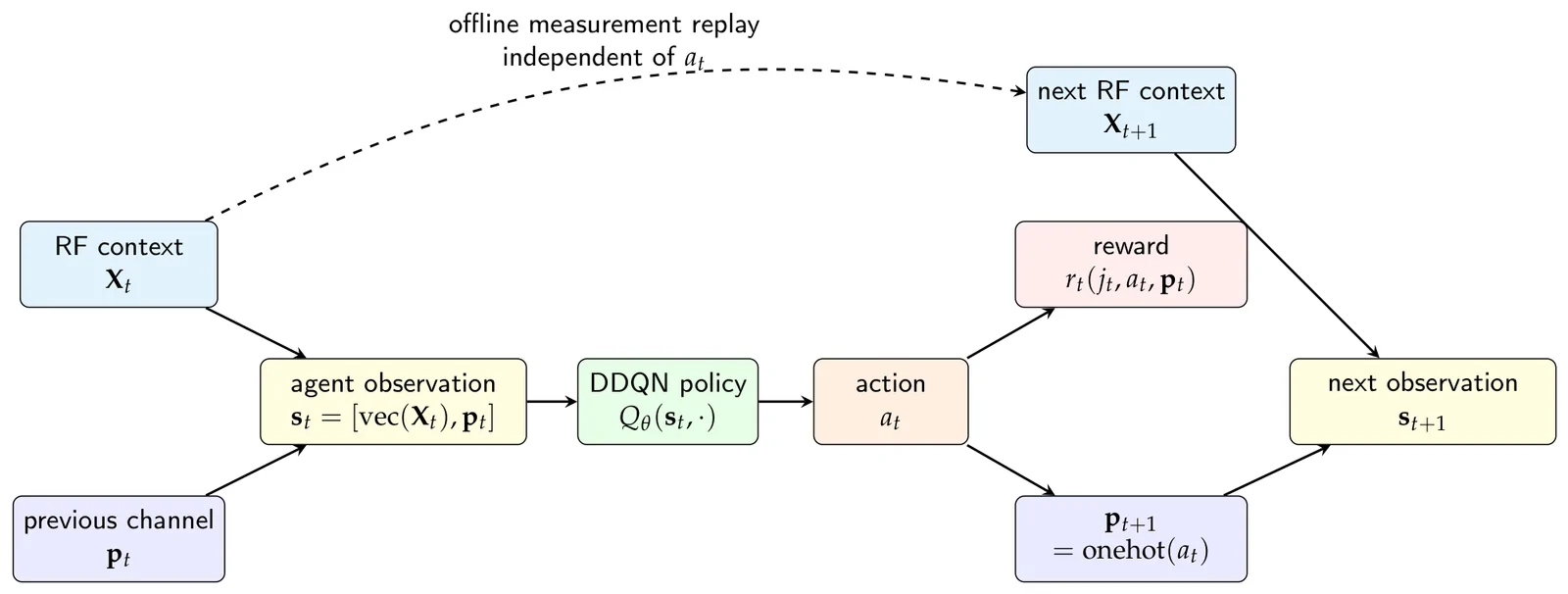
Switching-aware decision formulation. At step *t*, the observation received by the Double Deep Q-Network (DDQN) policy contains the exogenous radio-frequency (RF) summary $X_{t}$ and the previous-channel encoding. The action $a_{t}$ determines the selected channel and becomes the previous-channel component at t+1, but it does not alter the exogenous RF transition $P(X_{t+1}|X_{t})$.

Reinforcement learning commonly models sequential decisions as a Markov decision process (MDP) [14]. Such a formulation, however, must identify the part of the transition that is actually affected by an action. In the present spectrum-selection task, the RF measurement context evolves exogenously, whereas the selected channel is carried into the next observation and changes whether a later action incurs a switching cost. Bellman-style updates can represent this switching-dependent return [15], but their practical value must be tested against contextual and deterministic decision rules. Figure 2 separates the exogenous RF transition from the action-dependent previous-channel state, which is the source of switching-aware temporal coupling in the implemented problem.

Early model-free methods, including temporal-difference (TD) learning and Q-learning, were introduced by Sutton et al. [16] and Watkins et al. [17], respectively. Their tabular representations are effective in small state–action spaces but scale poorly as dimensionality increases. Function approximation addresses this limitation by replacing explicit value tables with parameterized models. A deep Q-network (DQN) combines Q-learning with deep neural networks for high-dimensional sensory inputs [18]; a Double Deep Q-Network (DDQN) reduces overestimation bias [19]; the Dueling architecture separates state-value and action-advantage estimation [20]; prioritized experience replay (PER) improves sampling efficiency [21]; and Rainbow integrates several such enhancements within a unified agent [22]. Despite these advances, deep reinforcement learning (DRL) still faces practical challenges in training stability, sample efficiency, hyperparameter sensitivity, and interpretability, particularly in engineering systems with limited computational and experimental resources.

Table 1 summarizes the established value-learning components used to contextualize, rather than by itself validate, the proposed function approximator.

A substantial body of work has applied RL and DRL to radar, communication, and spectrum-decision problems. DQN-based methods have been used for radar jamming and frequency selection as the number of states or channel options increases. This body of work includes the adaptive-radar studies by Liu et al. [8], Li and Dong [9], and Jiang et al. [11] together with the partially observable MDP study by Xing et al. [23]. DRL has also been applied to dynamic spectrum access, where model-free agents learn channel selection and access policies without explicit jammer models [24], as well as to power control, beam selection, and joint waveform–resource allocation [25,26,27]. Field- and simulation-based studies report improved adaptability over fixed-rule baselines while also revealing limited generalization when training conditions do not capture the diversity of adversarial behavior [28,29]. Recent bedrock-model research further examines generalization and transferability in communication and sensing through modular mathematical structures and parameter transfer across tasks [30].

In parallel with neural methods, polynomial bases and other structured function approximators have been used in approximation, control, system identification, and value-function estimation [31]. Their main advantage lies in explicit feature construction using compact low-order nonlinear terms. In value-based RL, Q-functionals employ basis functions, including polynomial bases, to represent action-dependent Q-value variation in continuous-control settings [32]. In contrast, the hybrid neural–polynomial deep Q-network (HNP-DQN) evaluated here applies an element-wise first- and second-order expansion to a learned latent observation representation before estimating eight discrete action values. This combination retains the flexibility of learned features while introducing an explicit low-order inductive bias suited to compact spectrum-observation representations [33]. The architecture permits a descriptive audit of the raw outgoing weights associated with its linear and squared column blocks. However, those magnitudes are scale dependent and do not quantify branch use, feature importance, causal contribution, or policy performance. Channel switching is also relevant to adaptive spectrum selection because frequent retuning can introduce synchronization, reconfiguration, and continuity costs. The present reward therefore includes a fixed switching penalty as a simplified representation of this overhead. Related studies have similarly incorporated switching costs into Q-learning-based channel selection and constrained contextual-bandit waveform selection [34,35]. Together, these considerations motivate evaluating the proposed hybrid architecture with a switching-aware reward in the measurement-driven decision setting considered in this paper.

For the present evaluation, three distinct sources of apparent policy advantage must be addressed: access to stronger interference information, model size, and genuinely action-dependent temporal value. This paper addresses these factors within one frozen measurement-replay protocol by declaring comparator information access, closely matching trainable parameter count between the neural models, and testing long-horizon learning against myopic and deterministic alternatives. This joint control strategy, rather than a universal superiority claim, defines the central evaluation contribution of this paper.

### 1.3. Objectives and Contributions

This paper evaluates HNP-DQN as a structured value-function approximator for a controlled, measurement-driven spectrum-selection layer. The contribution is limited to the eight-channel RF decision task studied here and is not presented as an end-to-end cognitive-radar or intelligent-jammer demonstration.

1. A hybrid neural–polynomial Q-network is developed by combining a learned latent representation with an explicit element-wise second-order expansion $\left[h,h^{⊙2}\right]$, layer normalization (LayerNorm), and a dueling Q-value head. The mapping expands representation width through linear and squared latent responses without explicit cross-feature products. A conventional MLP-DDQN with a closely matched trainable parameter count and separate single-component ablations are included to reduce parameter-count confounding when examining architectural structure. Across the six predeclared comparisons between HNP-DQN and the matched MLP, the return differences (HNP-DQN minus MLP) ranged from −1.121 to 0.388, every 95% confidence interval included zero, and the Holm-adjusted *p*-values ranged from 0.500 to 0.789. Thus, at the present statistical resolution, the approximately parameter-matched results did not distinguish the held-out return of HNP-DQN from that of the similarly sized nonlinear approximator. This matched-information comparison provides a rigorous architectural reference that reduces parameter-count confounding, but it should not be interpreted as a one-factor isolation of the squared-feature branch. HNP-DQN and the matched MLP used 32,841 and 32,691 trainable parameters, respectively. In the engineering profile, HNP-DQN had median central processing unit (CPU) and graphics processing unit (GPU) batch-1 latencies of 0.118 and 1.403 ms, respectively, and a median training time of 79.554 s; the corresponding values for the matched MLP were 0.084 ms, 1.159 ms, and 47.529 s. These timing summaries are descriptive engineering measurements rather than policy-performance inference.
2. The agent observation combines 40 RF summaries with an eight-dimensional previous-channel encoding. This exposes the only action-dependent transition component: the action does not change future RF measurements, but it changes whether a future decision incurs the fixed normalized switching cost $C_{\mathrm{switch}}=0.1$. This coefficient is a shared decision-level design choice; it is not a hardware-calibrated or universally optimal cost.
3. A frozen file-level protocol separates model development from evaluation. Under the 20 cm/10 dBm development condition, scan files 0–6 are used for training, file 7 is used for validation, and files 8–9 are labeled as a previously inspected within-condition pilot. The 40 cm/10 dBm and 20 cm/5 dBm configurations are outcome-uninspected evaluation-only distance and power transfers, which are declared by file names before any policy outcome was computed. Strong deterministic rules and the capacity-matched MLP are evaluated on common trajectories. Inference uses independently trained seeds, paired effect estimates, confidence intervals, and Holm correction; episodes are not treated as independent replicates.

Together, these elements provide an information-aware, approximately parameter-matched, and statistically reproducible evaluation of structured value learning. The results identify a conditional pattern rather than a universal algorithm ranking. Under deterministic sweeping, the schedule-aware rule uses the declared initial phase and one-channel-per-step direction and maintains an internal step counter to track the deterministic progression; these schedule variables and the corresponding step index are not included in HNP-DQN’s observation. Its superior return therefore shows the operational advantage attainable when this known schedule specification is directly exploited rather than a matched-information architectural advantage. Under random jamming, the comparisons with the observation-based threshold rule were inconclusive after multiplicity correction, as were the six comparisons with the capacity-matched MLP. Consequently, the contribution comprises both the HNP-DQN architecture and a controlled evaluation framework that documents comparator information access, closely matches trainable parameter count for the neural control, and probes switching-dependent temporal utility. Within the declared testbed, this framework provides an empirical reference for deciding when explicit prior knowledge should be used directly and when additional learning complexity requires further evidence. Acquisition-session-disjoint and new-site evaluation, radar-band and hardware validation, reactive or learning-based jammers, hardware-calibrated switching costs, and causal feature attribution remain the next validation stages.

The remainder of this paper is organized as follows. Section 2 describes the measurement-driven environment, file-level data protocol, jamming patterns, 48-dimensional observation, action space, and reward. Section 3 presents HNP-DQN and the capacity-matched MLP control. Section 4 reports the deterministic and learned comparisons, seed-level statistical analysis, model cost, component ablations, and descriptive weight analysis. Section 5 summarizes the verified findings and limitations.

## 2. Dataset and Environment Modeling

### 2.1. RF Jamming Dataset

The RF Jamming dataset developed by Ali et al. is an experimental dataset specifically designed for radio frequency interference (RFI) research. It was constructed using wireless spectrum scanning techniques and provides a standardized benchmark for studies on interference detection, interference mitigation, and anti-jamming strategies [36]. During data acquisition, the researchers employed a dedicated RFI monitoring platform that integrates the spectrum sensing capability of wireless network interface cards with the JamRF interference toolkit. This platform enables the controlled generation of diverse interference scenarios in laboratory environments while systematically recording radio frequency spectrum measurements.

The experimental platform was built around the Raspberry Pi Compute Module 4 and incorporates two widely used wireless modules, Qualcomm Atheros QCA9880 and Doodle Labs AR958x, for spectrum sensing and scanning. Interference signals were generated using a HackRF One software-defined radio in conjunction with the JamRF toolkit. Data collection was conducted in three representative environments, including a radio-frequency-shielded enclosure, a laboratory environment, and a typical office setting. During the experiments, the jamming transmission power was configured at three levels: 0 dBm, 5 dBm, and 10 dBm. The separation distance between the transmitter and receiver was varied among 20 cm, 40 cm, and 60 cm. Measurements were collected across both the 2.4 GHz and 5 GHz frequency bands. The spectrum-scanning configurations included the 20 and 40 MHz high-throughput modes (HT20 and HT40) and the 80 MHz very-high-throughput mode (VHT80). Consequently, the dataset captures a wide range of operating conditions involving different jamming intensities and channel environments.

Each raw record contains eight physical-layer features (frequency, noise, maximum amplitude, total gain, base power, received signal strength indicator (RSSI), relative power, and average power) extracted from the original spectrum scan headers. The raw records in comma-separated values (CSV) format additionally provide an snr field generated during the original dataset preprocessing. The present implementation reads this field directly; it is not recomputed from RSSI and noise and is not identified with a separately calibrated physical signal-to-noise ratio (SNR) or signal-to-interference-plus-noise ratio (SINR). We therefore use the term dataset-provided snr feature. Its empirical direction with respect to jammer occupancy is checked on development data before the maximum-quality/minimum-interference rule is defined: across the 56 training source files, the jammed channel’s window-level snr_mean averaged 27.626, whereas the non-jammed channel mean averaged −0.925. The channel-wise maximum identified the recorded jammer in 99.894% of training windows, while the minimum did so in 0.000%. We therefore define observable quality as −snr_mean; this is a corpus-specific direction check rather than a reinterpretation of calibrated physical SNR.

The dataset provides the measurement source for a controlled multi-channel RF spectrum-selection testbed. We select the 5 GHz band with eight consecutive 20 MHz sub-channels spanning 5180–5320 MHz. The acquisition hardware is a wireless spectrum scanner rather than radar hardware. Accordingly, the testbed supports a controlled spectrum-decision-layer evaluation motivated by radar spectrum agility; it does not validate detection, tracking, waveform, or hardware performance.

Measurements are split at the raw CSV scan-file level before window construction. Under the 20 cm/10 dBm development configuration, scan identifiers (IDs) 0–6 are used for training and scan ID 7 is used for validation. Scan IDs 8–9 were inspected in a pre-protocol rule-based engineering diagnostic and are therefore reported only as an explicitly labeled within-configuration pilot rather than a confirmatory test. Two outcome-uninspected cross-configuration evaluations were declared by file names before any policy outcome was computed: all scan IDs 0–9 at 40 cm/10 dBm form a distance shift, and all scan IDs 0–9 at 20 cm/5 dBm form a power shift. Files could be loaded for format and finite-value audits, but these audits did not compute or inspect policy outcomes. Neither the pilot nor either transfer condition is used for threshold fitting, hyperparameter selection, early stopping, or architecture changes. Table 2 records these fixed roles and window counts; the file-level separation prevents a raw row from entering more than one development split.

Within each raw file, a 32-sample window is generated with stride 1. Windows never cross file boundaries, and no raw row contributes to more than one development split. Each window is summarized by the mean and standard deviation of the dataset-provided snr feature, the RSSI mean and standard deviation, and the mean noise level for each channel. These 40 RF summaries are combined with the previous-channel encoding in Section 2.4.

**Figure 3:**
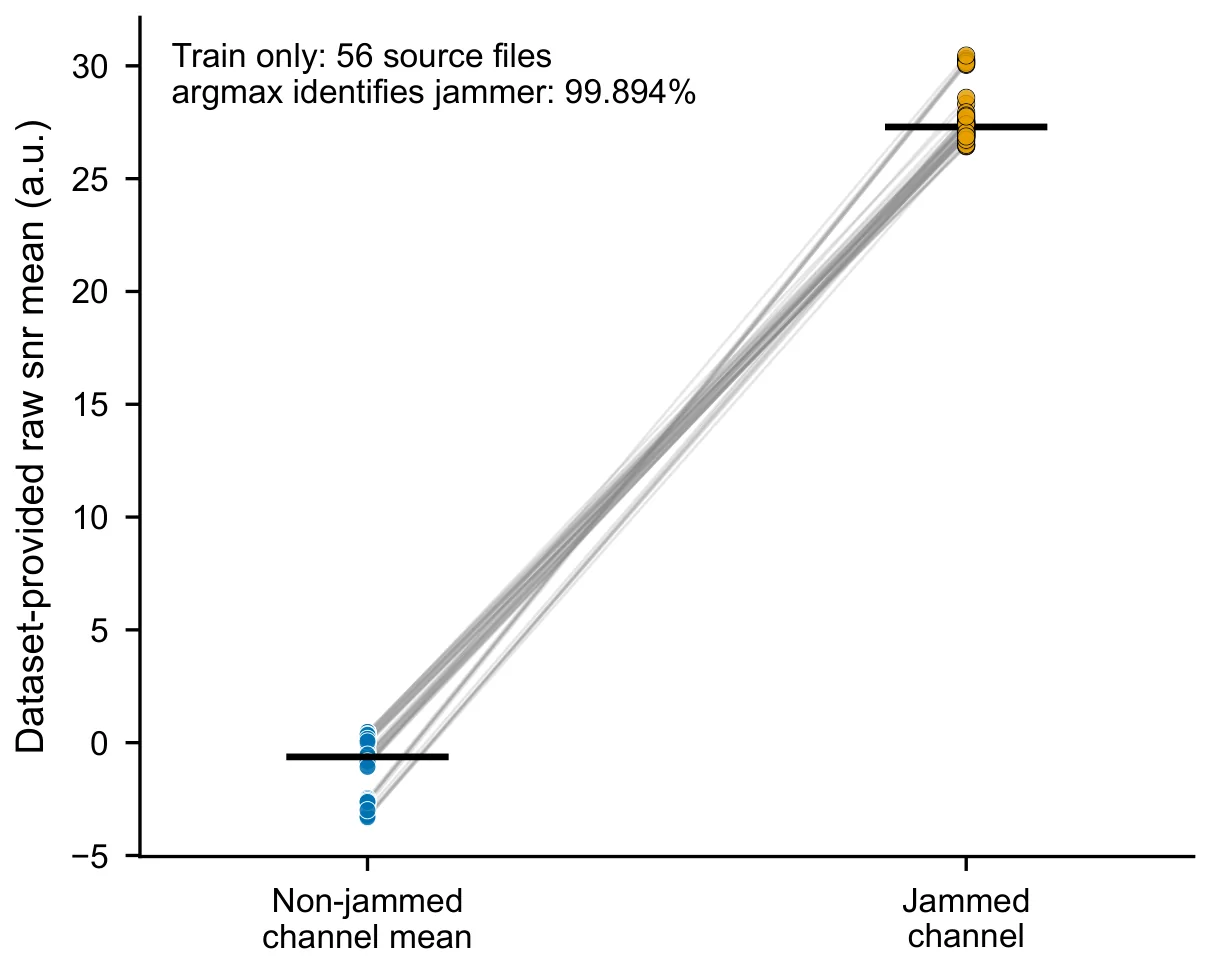
Development-training-only audit of the dataset-provided snr field. Each paired line represents one of 56 fixed-jammer source files from scan identifiers (IDs) 0–6. Points show the within-file mean of the jammed channel and the mean over its seven non-jammed channels; horizontal bars denote medians. The channel with the largest raw snr_mean matched the recorded jammer label in 99.894% of training windows. Accordingly, the observable quality rule maximizes −snr_mean (equivalently, minimizes the raw field). No pilot or transfer condition was used for this direction audit.

The same dataset-provided field contributes its windowed mean and standard deviation to the RF observation together with the RSSI mean, RSSI standard deviation, and mean noise. Because its chipset-level derivation is unavailable, we do not equate it with the physical SINR defined below. Figure 3 provides the training-only sign audit: the channel with the largest raw snr_mean matched the recorded jammer label in 99.894% of the audited training windows without using pilot or transfer outcomes.

### 2.2. Radio-Frequency Environment Model

We model the radar-oriented, spectrum-decision-layer frequency-selection problem using a communication-system RF measurement testbed and a discrete spectrum environment. The operating spectrum is represented as a finite set of non-overlapping frequency channels, which is denoted by

(1) $$C=\{f_{1},f_{2},\ldots ,f_{N}\},$$

where each element corresponds to a potential carrier frequency that the agent may occupy. In the evaluated setting, N=8 and $f_{i}=5180+20\left(i-1\right)$ MHz. This discrete representation captures the sensing–decision–adaptation loop required for interference-aware frequency selection while retaining a compact action space for controlled algorithm comparison.

For physical interpretation, the received power on channel *c* at decision step *t* can be decomposed in the linear-power domain as

(2) $$P_{\mathrm{tot}}\left(c,t\right)=P_{S}\left(c,t\right)+P_{J}\left(c,t\right)+P_{N}\left(c,t\right),$$

where $P_{S}\left(c,t\right)$ is the received useful-signal power, $P_{J}\left(c,t\right)$ is the received jammer power, and $P_{N}\left(c,t\right)$ is the background noise power. The corresponding physical signal-quality ratios are

(3) $${\mathrm{SNR}}_{\mathrm{phy}}\left(c,t\right)=\frac{P_{S}\left(c,t\right)}{P_{N}\left(c,t\right)},\;{\mathrm{SINR}}_{\mathrm{phy}}\left(c,t\right)=\frac{P_{S}\left(c,t\right)}{P_{J}\left(c,t\right)+P_{N}\left(c,t\right)}.$$

Power components must be added in the linear domain. For *P* expressed in milliwatts, $P\left[\mathrm{dBm}\right]=10\log_{10}\left(P\left[\mathrm{mW}\right]\right)$, whereas a dimensionless power ratio *R* is represented as $R\left[\mathrm{dB}\right]=10\log_{10}R$. Therefore, the total received power in dBm, an SNR in dB, and an SINR in dB refer to different quantities.

The equations above provide a physical interpretation of signal, interference, and noise. The implemented environment does not reconstruct these quantities; it uses the fields contained in the RF Jamming dataset. In particular, the raw records provide a dataset-provided snr feature together with RSSI and noise. The snr field is distinct from the theoretical SINR above and is not described as a calibrated physical ratio.

For each channel *i* and decision step *t*, process_data extracts a 32-sample measurement window with stride 1 within a single raw scan file. The environment summarizes the window using the mean and standard deviation of the dataset-provided snr feature, the mean and standard deviation of RSSI, and the mean noise level. Stacking the five statistics from all eight channels yields an 8×5 feature matrix, which is flattened into a 40-dimensional RF context. Values of the dataset-provided snr feature outside [−100,100] are treated as invalid before aggregation. The 32 raw samples are therefore summarized through statistical aggregation rather than concatenated into a 256-dimensional history. Window generation occurs after the file-level split, and windows never cross file or split boundaries.

Each episode begins from a seeded window index, a jammer position generated by the selected jammer rule, and a reset previous-channel indicator. Let $z_{t}$ denote the simulator-internal replay state: physical condition, scan ID, ordinal window index, jammer-rule state, and random-generator state. The environment constructs $X_{t}$ from $z_{t}$ and combines it with the previous-channel encoding to form $s_{t}$. After the agent selects $a_{t}$, collision and reward are evaluated against the current jammer. With $\xi_{t}$ denoting exogenous random input, the generator uses

(4) $$z_{t+1}=F\left(z_{t},\xi_{t}\right),\;X_{t+1}=G\left(z_{t+1}\right),$$

where neither *F* nor *G* receives $a_{t}$. By contrast, the observed previous-channel component satisfies

(5) $$p_{t+1}=\mathrm{onehot}\left(a_{t}\right).$$

The action therefore changes current reward and future switching dependence, but it is not an input to the exogenous measurement-replay generator.

Each raw source file was recorded under one fixed jammer label. Within a file, the eight frequency records occur as consecutive blocks; no timestamp verifies that observations assigned the same ordinal index across these blocks are concurrent. To construct a sweeping or random episode, the measurement-replay simulator selects the file associated with the current jammer label and advances a shared ordinal window index. The source files likewise provide no verified cross-file timestamps. Consequently, neither the 40-value multi-channel context nor adjacent simulated observations are claimed to be time-synchronized physical measurements. Sweeping and random refer to the simulator’s jammer-label schedule. The resulting observations and transitions are offline, synthetic replay constructions assembled from measured within-file records.

Training uses only the declared 20 cm/10 dBm training files, whereas model and threshold selection use only the corresponding training and validation files. The pre-inspected pilot and both evaluation-only transfer configurations use fixed, common trajectories after the policy and analysis code are frozen. The evidence therefore concerns file-disjoint within-configuration performance and transfer to the two declared distance/power shifts; it is not evidence of acquisition-session-disjoint or new-site generalization.

### 2.3. Jamming Patterns

The environment considers two complementary interference patterns: periodic sweeping and independent random jamming. These label schedules provide controlled deterministic and stochastic conditions for evaluating policy behavior in the measurement-replay simulator. Each jammer operates within the same discrete frequency set C defined in Section 2.2 and occupies a 20 MHz interval centered at its current frequency. In the implemented discrete rule, collision occurs when the selected channel center satisfies

(6) $$\left|f_{a_{t}}-f_{j_{t}}\right|\leq \frac{B_{J}}{2},\;B_{J}=20\;\mathrm{MHz}.$$

Because adjacent candidate centers are separated by 20 MHz, the rule identifies the channel centered at the current jammer frequency as collided. The corresponding reward and switching-cost cases are defined in Section 2.6.

**Figure 4:**
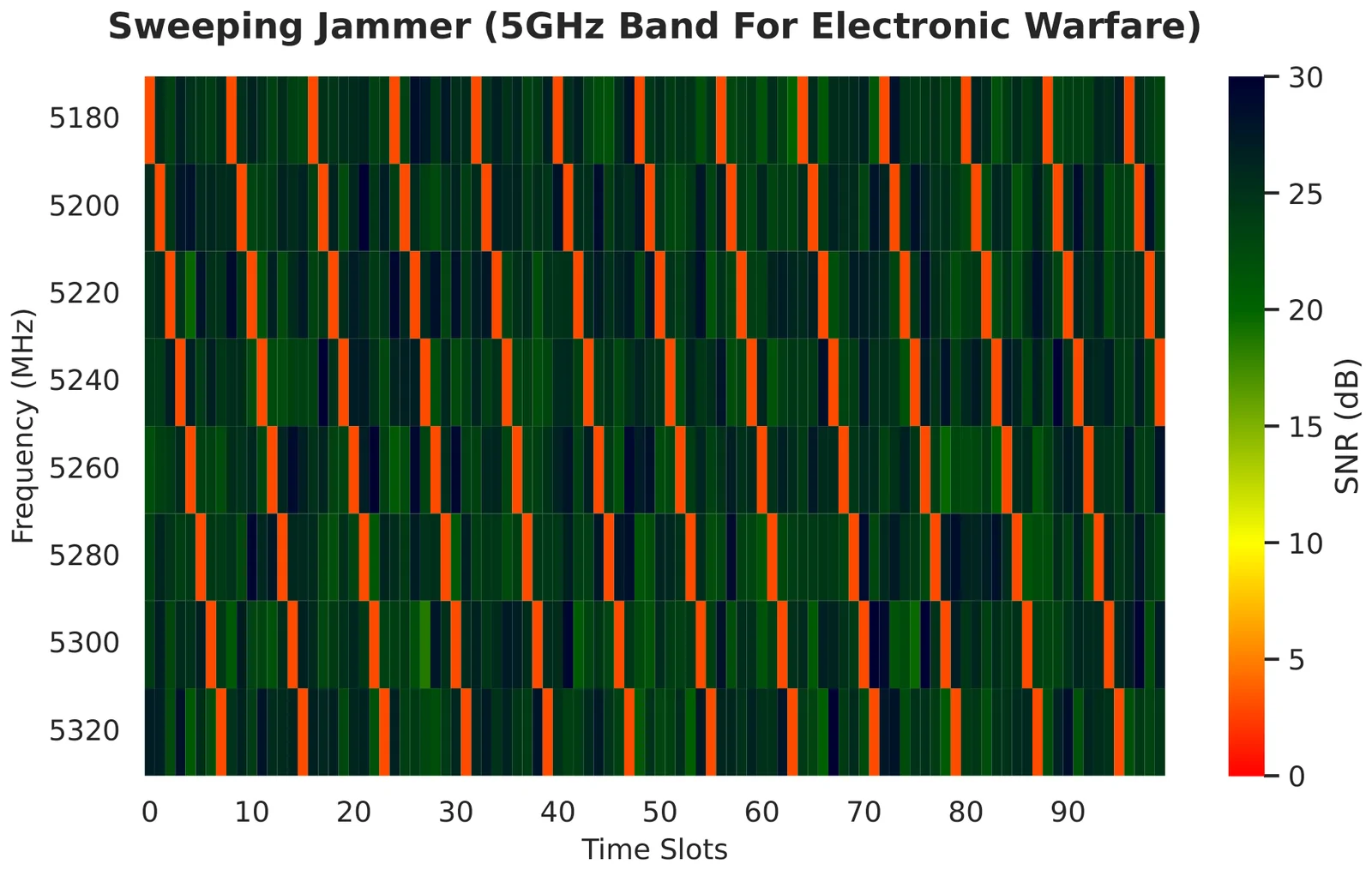
Time–frequency occupancy of the periodic sweeping jammer over the eight candidate channels.

The sweeping jammer periodically switches among the candidate channels with a fixed step size, forming a deterministic cyclic sequence. Figure 4 illustrates the jamming distribution across the 5180–5320 MHz frequency band over 100 decision steps. At the beginning of the sequence, the jammer occupies the first candidate channel. After the reward at step *t* is evaluated, the jammer advances by one channel, wraps around after the eighth channel, and generates the measurements used for $s_{t+1}$. The jammer therefore remains at one channel for one decision interval. This periodic structure provides a repeatable test of whether the policy can exploit regularity already reflected in the sensed multi-channel observation while maintaining low switching overhead.

**Figure 5:**
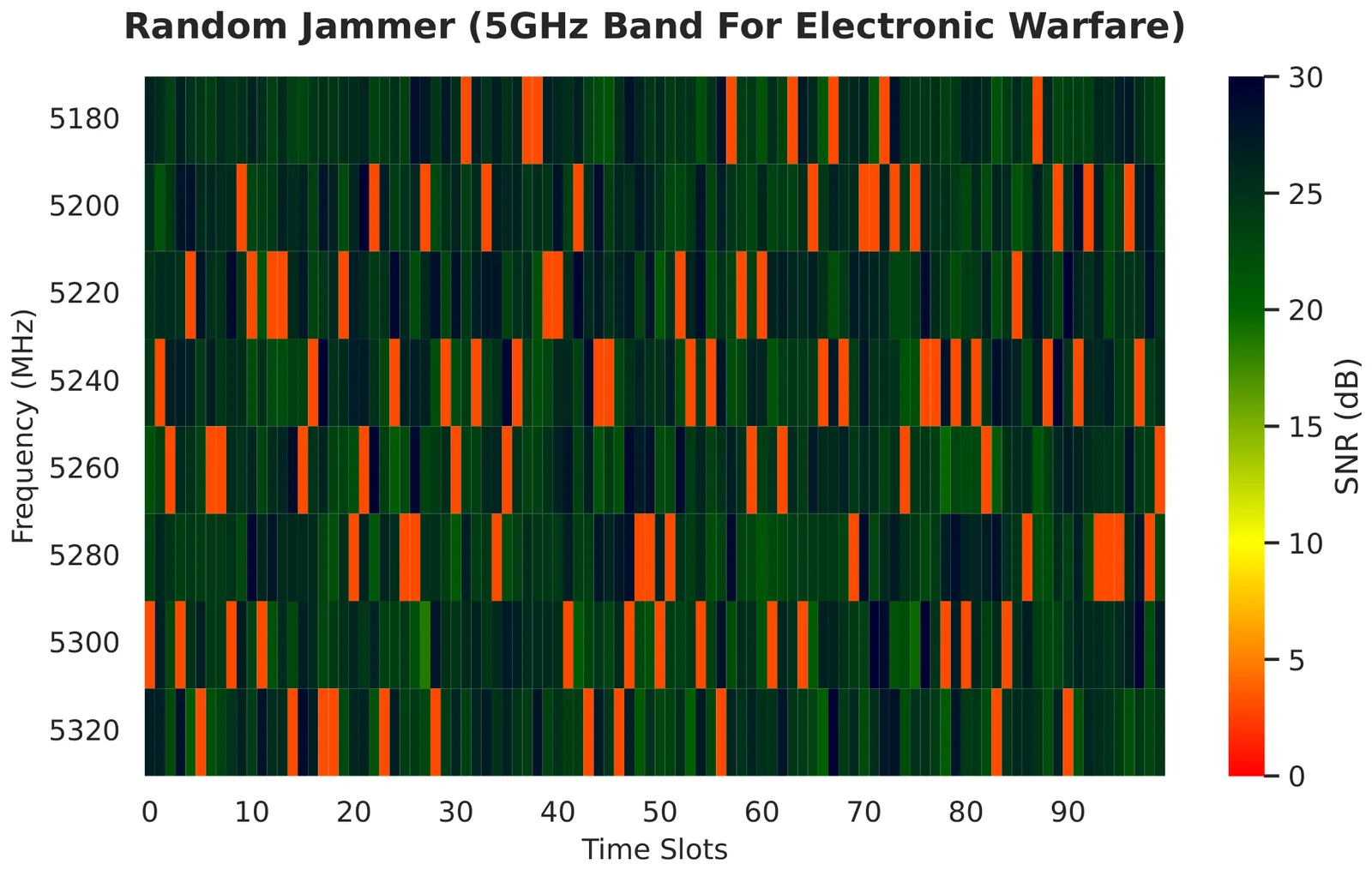
Time–frequency occupancy of the independent random jammer over the eight candidate channels.

The random jammer independently and uniformly selects a channel from the candidate channel set at each time slot to impose jamming. Figure 5 illustrates the jamming distribution over the same time and frequency range. The current random jammer position determines $s_{t}$ and the reward for $a_{t}$. After the reward is evaluated, the next jammer index is drawn independently as

(7) $$j_{t+1}∼\mathrm{Uniform}\left\{1,2,\ldots ,N\right\},\;N=8.$$

The next observation is then generated from the newly selected interference condition and the next measurement window. In contrast to sweeping interference, this process has no deterministic channel progression. It therefore complements the periodic case by evaluating policy behavior under slot-wise stochastic spectrum occupancy. Together, the two patterns cover structured and random interference within the controlled eight-channel measurement domain.

### 2.4. Agent Observation Representation

The adaptive spectrum-selection task is represented as a switching-aware sequential decision process. We use Bellman-style function approximation but do not assume that the retained rolling summaries have been proven to be a sufficient Markov state for the underlying RF process. The evaluated environment contains N=8 channels with center frequencies $f_{i}=5180+20\left(i-1\right)$ MHz. At each decision step, the observation combines the measured condition of all candidate channels with the channel selected at the preceding step. The RF context is exogenous, whereas the previous-channel component is action dependent.

Let $W_{i,t}$ denote the within-file measurement window of channel *i* at decision step *t*. Five statistics are calculated from the dataset-provided snr, RSSI, and noise fields:

(8) $$x_{i,t}={\left[\mu_{\mathrm{SNR}}\left(W_{i,t}\right),\sigma_{\mathrm{SNR}}\left(W_{i,t}\right),\mu_{\mathrm{RSSI}}\left(W_{i,t}\right),\sigma_{\mathrm{RSSI}}\left(W_{i,t}\right),\mu_{\mathrm{noise}}\left(W_{i,t}\right)\right]}^{T}.$$

Here, the SNR subscript denotes the dataset-provided snr column rather than the physical SNR in Section 2.2. The channel-feature matrix is

(9) $$X_{t}={\left[x_{1,t},x_{2,t},\ldots ,x_{N,t}\right]}^{T}\in R^{N\times 5}.$$

Let $p_{t}\in {\left\{0,1\right\}}^{N}$ be a one-hot encoding of the channel selected at step t−1. At reset, before any action has been selected, $p_{1}=0$. For t>1,

(10) $$p_{t}=\mathrm{onehot}\left(a_{t-1}\right).$$

The agent input is

(11) $$s_{t}={\left[\mathrm{vec}{\left(X_{t}\right)}^{T},p_{t}^{T}\right]}^{T}\in R^{5N+N}=R^{48}.$$

The previous-action block makes the switching-dependent reward observable, but it does not by itself establish that the RF summaries are Markov-sufficient. All learned models and ordinary observation-based deterministic baselines receive the same 48-dimensional observation. The schedule-aware sweep rule is identified separately because it uses the declared initial phase and one-channel-per-step direction and maintains an internal step counter to track the deterministic progression; these schedule variables and the corresponding step index are not part of the 48-dimensional agent observation. The jammer-aware greedy reference may use the current simulated jammer label, while the clairvoyant dynamic-programming oracle may use its entire realized future sequence; neither privileged reference is presented as deployable.

The dataset-provided snr feature is used directly during environment construction. Values outside [−100,100] are treated as invalid before the window statistics are formed, and missing entries are completed only from valid measurements within the same window. In the development files, all eight noise_mean entries equal −102 and therefore become zero after the training-fitted standardization. This field is retained for schema compatibility but is not claimed to add independent predictive information in this dataset.

At step *t*, the agent receives $s_{t}$ under jammer position $j_{t}$ and selects $a_{t}$. Collision and reward are evaluated using the same $j_{t}$. Afterward, the jammer and within-file window advance exogenously, while $a_{t}$ is written into $p_{t+1}$. Consequently, an action affects the next previous-channel component and future switching-dependent reward. It does not, however, cause a change in the next RF measurement. This distinction is central to the problem formulation and to the comparison with myopic rules.

### 2.5. Action Space and Control Constraints

The action space comprises the transmission channels available to the agent at each time step and therefore matches the candidate channel set represented in the observation:

(12) $$A=\{a_{1},a_{2},\ldots ,a_{N}\},$$

where $a_{i}$ selects the *i*-th candidate channel and is mapped to the center frequency $f_{i}$. In the evaluated environment, N=8.

Because the previous channel is part of the agent observation, the switching indicator is defined as

(13) $$I_{t}^{\mathrm{sw}}=\left\{\begin{array}{cc} 0, & t=1,\\ I(a_{t}\neq a_{t-1}), & t>1. \end{array}\right.$$

At reset, $I_{1}^{\mathrm{sw}}=0$ and $p_{1}=0$; no episode inherits a selected channel, collision count, or step count from an earlier episode. The switching cost is a normalized decision-level proxy for continuity; it is not a measured hardware latency or energy cost.

### 2.6. Reward Design and Performance Objectives

The reward prioritizes interference avoidance and then uses the switching indicator to distinguish between two successful decisions. Let $f_{a_{t}}$ and $f_{j_{t}}$ denote the selected-channel and jammer center frequencies. Both the candidate-channel bandwidth and the jammer bandwidth are 20 MHz, and adjacent channel centers are separated by 20 MHz. Under this equal-width discrete grid, positive spectral overlap occurs only when the selected and jammed channels have the same center. The collision indicator is therefore written as

(14) $$I_{t}^{\mathrm{col}}=I\;\left(f_{a_{t}}=f_{j_{t}}\right).$$

Equivalently, the positive-overlap condition can be expressed as $|f_{a_{t}}-f_{j_{t}}|\;<\left(B_{\mathrm{ch}}+B_{\mathrm{jam}}\right)/2$ with $B_{\mathrm{ch}}=B_{\mathrm{jam}}=20$ MHz. The equality form above is used because it directly matches the present eight-channel decision grid.

The reward function is

(15) $$r_{t}=\left\{\begin{array}{cc} -1, & I_{t}^{\mathrm{col}}=1,\\ 1, & I_{t}^{\mathrm{col}}=0\;\mathrm{and}\;I_{t}^{\mathrm{sw}}=0,\\ 1-C_{\mathrm{switch}}, & I_{t}^{\mathrm{col}}=0\;\mathrm{and}\;I_{t}^{\mathrm{sw}}=1, \end{array}\right.\;C_{\mathrm{switch}}=0.1.$$

Consequently, a collision receives −1, successful retention receives 1, and a successful switch receives 0.9. A collision is treated as the dominant failure event, so the switching cost is not added to the collision penalty. This ordering preserves a clear decision priority: interference avoidance first, followed by channel continuity among successful actions.

The value $C_{\mathrm{switch}}=0.1$ is fixed before evaluation and is shared by all policies. It introduces a modest normalized continuity preference, but it is not optimized, hardware calibrated, or claimed to be universally appropriate. A comprehensive sensitivity study tied to measured retuning, energy, synchronization, or sensing-performance cost is outside the scope of this paper.

For an episode of T=100 steps, performance is measured primarily through the accumulated return

(16) $$G_{e}=\sum_{t=1}^{T}r_{t}.$$

For each independently trained seed, the primary endpoint is the mean 100-step evaluation return across the common fixed trajectories. Supporting endpoints are collision count/rate and switch count/rate. The independently trained seed, not an episode or step, is the inferential unit. For each comparison, the analysis reports every seed, the mean and standard deviation, the paired mean difference, its 95% confidence interval (CI), the paired standardized effect size, and a two-sided paired sign-flip/permutation test with Holm correction for the declared comparison family. The analysis included 10 independently trained seeds per learned method and jammer mode with each seed evaluated on the same 20 fixed trajectories per physical condition and jammer mode. Episode-level distributions, if shown, are labeled descriptive and are not used to establish significance.

## 3. HNP-DQN Architecture

### 3.1. Proposed Architecture

**Figure 6:**
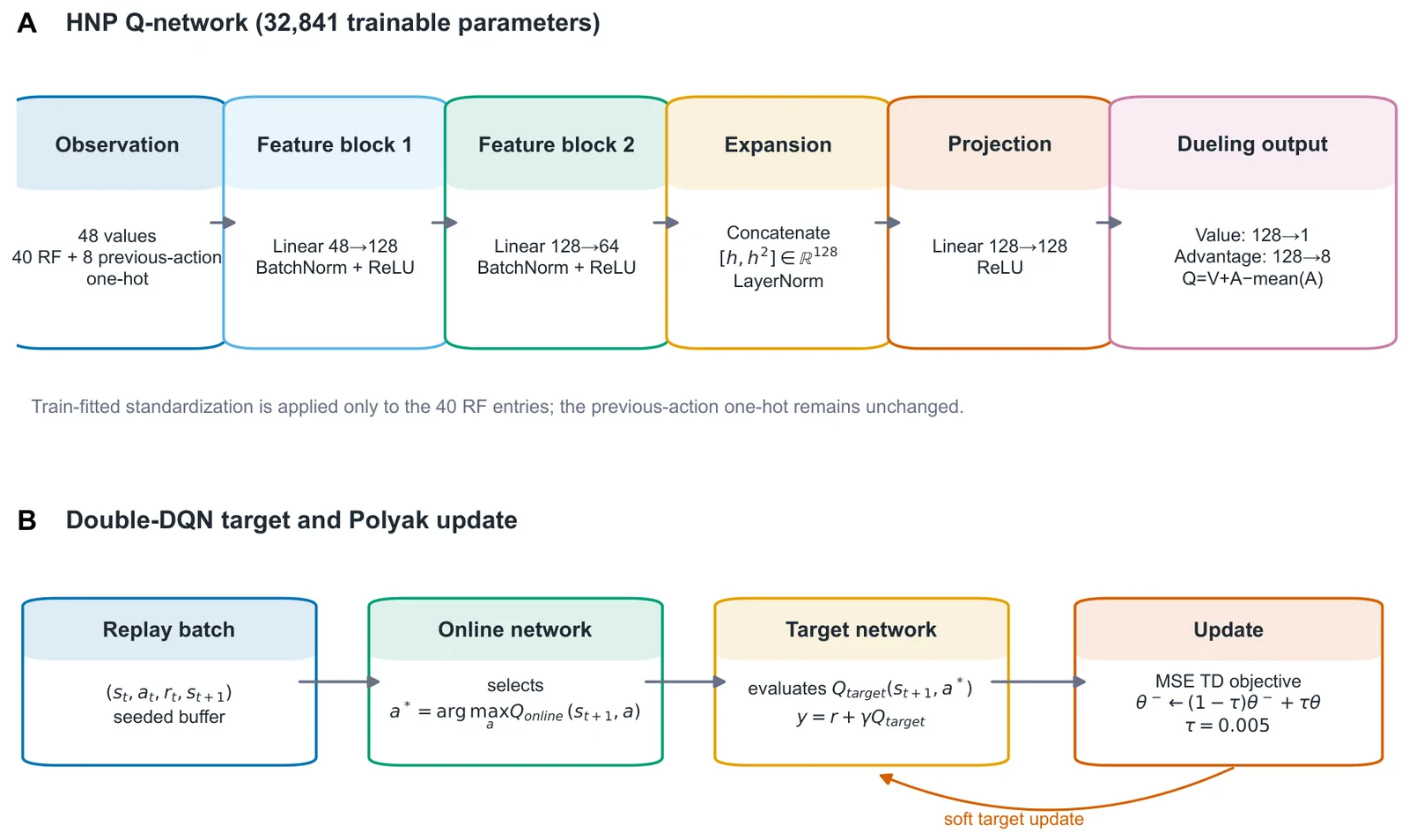
Frozen hybrid neural–polynomial deep Q-network (HNP-DQN) implementation. (**A**) The 48-dimensional observation contains 40 RF summaries and an eight-dimensional previous-action one-hot. Only the RF entries are standardized. The network maps 48→128→64, concatenates the latent vector and its element-wise square, applies layer normalization (LayerNorm) and a 128-dimensional projection, and uses dueling value and advantage heads. (**B**) DDQN uses the online network for next-action selection and the target network for evaluation. The loss uses a mean squared error (MSE) objective on the TD error, and target parameters follow the Polyak update with τ=0.005. The asterisk denotes the greedy next action selected by the online network, represented by $a^{*}$ in panel (**B**).

The agents learn channel action values from replay with a mean-squared TD objective: DQN uses one online network, DQN with Fixed Q Targets adds a target network, and DDQN selects actions online and evaluates them with the target network. In the diagram, BatchNorm denotes batch normalization (BN), and rectified linear unit (ReLU) is used as the activation function. Figure 6 distinguishes the feature pathway in panel A from the common DDQN update in panel B; the explicit polynomial expansion, rather than DDQN itself, is the component under study.

HNP-DQN combines a neural feature extractor with an explicit element-wise second-order expansion and LayerNorm. Its input is the 48-dimensional observation defined in Section 2.4: 40 RF summaries and an eight-dimensional previous-channel encoding. The width-expanded neural–polynomial representation is the component under study; dueling decomposition and the DDQN update are established value-learning components used as the common backbone.

The network contains three stages. First, the feature extractor maps the 48-dimensional observation through hidden widths 128 and 64. Second, the polynomial module concatenates the 64-dimensional latent vector with its element-wise square, applies LayerNorm, and projects the resulting 128-dimensional representation to a 128-dimensional feature vector. Third, separate value and advantage heads generate the final action values. The component ablations in Section 4 remove the polynomial expansion, LayerNorm, or dueling decomposition while retaining the remaining learning procedure.

#### 3.1.1. Feature Extraction Layer

Let $s\in R^{48}$ denote the switching-aware agent observation defined in Section 2.4. The feature extractor uses two fully connected layers, each followed by BN and ReLU activation:

(17) $$\begin{array}{cc} u & =\mathrm{ReLU}\left({\mathrm{BN}}_{1}\left(W_{1}s+b_{1}\right)\right),\\ h & =\mathrm{ReLU}\left({\mathrm{BN}}_{2}\left(W_{2}u+b_{2}\right)\right), \end{array}$$

where $W_{1}\in R^{128\times 48}$, $b_{1}\in R^{128}$, $W_{2}\in R^{64\times 128}$, and $b_{2}\in R^{64}$. The resulting latent representation is $h\in R^{64}$. This shallow neural stage learns mixtures of the dataset-provided snr, RSSI, noise, and previous-channel features before the explicit polynomial transformation. Consequently, the latent coordinates are learned features and are not in one-to-one correspondence with physical frequency channels.

#### 3.1.2. Polynomial Mapping Layer

The polynomial mapping is the main structural addition to the value network. It expands each learned latent coordinate using its first- and second-order element-wise terms. The operation does not include products between different latent coordinates and therefore does not explicitly encode cross-feature interactions. It is used as a controlled width expansion with an explicit second-order feature map. This construction makes the tested mapping explicit, but it does not by itself make branch contributions interpretable or causal.

(18) $$z={\left[h_{1},\ldots ,h_{64},\;h_{1}^{2},\ldots ,h_{64}^{2}\right]}^{⊤}\in R^{128},$$

where the first 64 entries retain the learned latent features and the remaining 64 entries contain their element-wise squares. No constant term or $h_{i}h_{j}$ term for i≠j is included. Including all pairwise cross-feature terms would increase the expansion from 128 dimensions to 2144 dimensions for the 64-dimensional latent representation and substantially increase the projection-layer parameters and computational cost. The adopted element-wise expansion is therefore a restricted structural choice rather than an exhaustive polynomial basis. The expansion itself is deterministic and contains no learned polynomial coefficients; any capacity or efficiency advantage must be established against the matched MLP and the implementation-cost results in Section 4.

LayerNorm is applied immediately after expansion to normalize the first- and second-order terms within each sample:

(19) $$\tilde{z}=\mathrm{LayerNorm}\left(z\right),$$

(20) $$\mu =\frac{1}{D}\sum_{k=1}^{D}z_{k},\;\sigma^{2}=\frac{1}{D}\sum_{k=1}^{D}{\left(z_{k}-\mu \right)}^{2},$$

(21) $${\tilde{z}}_{k}=\gamma_{k}\cdot \frac{z_{k}-\mu }{\sqrt{\sigma^{2}+\epsilon }}+\beta_{k},$$

where D=128, and $\gamma ,\beta \in R^{128}$ are the learnable affine parameters of LayerNorm. The normalized vector is then processed by a trainable projection and a ReLU activation:

(22) $$z^{\prime }=\mathrm{ReLU}\;\left(W_{\mathrm{proj}}\tilde{z}+b_{\mathrm{proj}}\right),\;z^{\prime }\in R^{128},$$

where $W_{\mathrm{proj}}\in R^{128\times 128}$ and $b_{\mathrm{proj}}\in R^{128}$. The trainable parameters associated with the expanded representation are therefore located in LayerNorm, the projection layer, and the downstream Q-value heads rather than in the expansion operation itself. Section 4.6 further analyzes $W_{\mathrm{proj}}$ to examine how the learned projection weights are distributed across the first- and second-order expanded representation.

#### 3.1.3. Q-Value Decomposition Module

The 128-dimensional vector $z^{\prime }$ is supplied to the dueling value and advantage heads. The dueling decomposition is an established component included to separate the shared state-value estimate from action-dependent deviations:

(23) $$V\left(s\right)=w_{V}^{⊤}z^{\prime }+b_{V},\;A\left(s,a\right)=w_{A,a}^{⊤}z^{\prime }+b_{A,a},$$

where $w_{V}\in R^{128}$ and $w_{A,a}\in R^{128}$. The value head outputs one scalar, whereas the advantage head outputs one value for each of the eight candidate actions. Mean advantage normalization resolves the identifiability ambiguity:

(24) $$Q\left(s,a\right)=V\left(s\right)+A\left(s,a\right)-\frac{1}{\left|A\right|}\sum_{a^{\prime }=1}^{\left|A\right|}A\left(s,a^{\prime }\right).$$

This aggregation is implemented directly in the forward pass of HNP-DQN. The contribution of the dueling head is evaluated by the No-Dueling variant in Section 4 rather than assumed from the architecture alone.

### 3.2. Capacity-Matched Conventional Control

To reduce parameter-count confounding when comparing the explicit expansion with a conventional neural alternative, the evaluation includes an approximately parameter-matched MLP-DDQN selected using only the training and validation files. It receives the same 48-dimensional observation and uses the same DDQN target, dueling aggregation, optimizer, replay buffer, target update, exploration schedule, update budget, seeds, and evaluation trajectories as HNP-DQN. It contains no explicit $\left[h,h^{⊙2}\right]$ mapping. Its hidden widths and exact parameter count are 48→174→128 with BatchNorm and ReLU after both hidden layers, which are followed by the same dueling head. HNP-DQN contains 32,841 trainable parameters and the matched MLP contains 32,691, which is an absolute difference of 150 parameters (0.46% relative to HNP-DQN).

Here, “capacity-matched” denotes the close matching of trainable parameter count rather than one-factor architectural matching. The comparator preserves the dueling head, but it also has different hidden widths and no post-expansion LayerNorm. It therefore reduces parameter-count confounding without by itself identifying a causal effect of the squared terms. The matched comparison is interpreted jointly with the narrower No-Polynomial and No-LayerNorm single-component ablations as a triangulated architectural check.

### 3.3. Training and Target-Update Procedure

HNP-DQN is trained with uniform experience replay, a DDQN bootstrap target, a softly updated target network, and gradient clipping.

The experience replay mechanism stores the transition samples $\left(s_{t},a_{t},r_{t},s_{t+1},d_{t}\right)$ generated through agent–environment interaction in a replay buffer D with capacity $N_{D}$, from which mini-batches of size *B* are uniformly sampled during training for parameter updates.

(25) $$L\left(\theta \right)=E_{(s,a,r,s^{\prime },d)∼D}\left[{\left(Q_{\theta }\left(s,a\right)-y^{\mathrm{DDQN}}\right)}^{2}\right].$$

For each learning update, the implementation computes the mini-batch mean-squared TD error. This quantity is used as an optimization diagnostic. Since DQN, DQN with Fixed Q Targets, and DDQN construct their bootstrap targets differently, absolute TD-loss magnitudes are not treated as direct, cross-model measures of policy quality or convergence speed.

The bootstrap target used by HNP-DQN is the standard DDQN target:

(26) $$y^{\mathrm{DDQN}}=r+\gamma \left(1-d\right)Q_{\theta^{-}}\;\left(s^{\prime },\underset{a^{\prime }}{\mathrm{argmax}}Q_{\theta }\left(s^{\prime },a^{\prime }\right)\right),$$

where the online network selects the next action and the target network evaluates that action. The complete target computation is performed without gradient tracking, so no gradient is propagated through $y^{\mathrm{DDQN}}$. The implemented target is exactly the expression above; it is not interpolated with the current prediction $Q_{\theta }\left(s,a\right)$.

The target-network parameters are updated after each learning iteration using a Polyak soft update:

(27) $$\theta^{-}\leftarrow \tau \theta +\left(1-\tau \right)\theta^{-},$$

where τ controls how quickly the target network tracks the online network.

Gradient clipping constrains the Euclidean norm of the online-network gradient before the Adam update:

(28) $$g=\nabla_{\theta }L\left(\theta \right)\cdot \frac{\kappa }{\max\;\left({∥\nabla_{\theta }L\left(\theta \right)∥}_{2},\kappa \right)},$$

with κ=0.7 in the evaluated configuration. Gradient clipping is applied throughout training as a fixed norm-control step; its use is not itself evidence of superior convergence or policy stability.

With respect to the exploration strategy, an ε-greedy policy is employed to balance exploration and exploitation during training:

(29) $$a_{t}=\left\{\begin{array}{cc} \mathrm{random}\;\mathrm{action}\;\mathrm{from}\;A, & \mathrm{with}\;\mathrm{probability}\;\varepsilon_{t},\\ {\mathrm{argmax}}_{a\in A}Q_{\theta }\left(s_{t},a\right), & \mathrm{with}\;\mathrm{probability}\;1-\varepsilon_{t}, \end{array}\right.$$

where $a_{t}$ is the selected action, A is the action space, and $Q_{\theta }\left(s_{t},a\right)$ is the action-value function.

The exploration rate is updated once after each episode according to the reciprocal multiplicative-decay rule

(30) $$\varepsilon_{e}=\max\left(\varepsilon_{\min},\frac{1}{1+e/\lambda_{\varepsilon }}\right),$$

where *e* is the zero-based episode number, $\varepsilon_{\min}=0.01$, and $\lambda_{\varepsilon }=10$ is the frozen episode-denominator value. Thus, exploration starts from 1.0 and decreases once per episode; over the 200-episode training horizon, the reciprocal term remains above the lower bound of 0.01.

### 3.4. Agent Training Procedure

The complete learning process is organized into 200 episodes, each containing at most 100 interaction steps. At each step, the agent selects an action using the ε-greedy policy, executes it, receives the reward and next observation, and stores the transition in the replay buffer. When a learning update is performed, a mini-batch is sampled to optimize the online network, after which the target network is updated using the Polyak soft-update rule. The exploration rate is updated after each episode according to the schedule defined above.

The 100-step boundary is implemented as a time-limit truncation rather than an absorbing environmental terminal state. Data collection stops at the boundary, but the final stored transition retains the DDQN bootstrap mask. This avoids treating remaining horizon as an unobserved terminal-state variable in the 48-dimensional observation.

## 4. Experiments and Results Analysis

### 4.1. Experimental Setup and Parameter Configuration

The final software and hardware configuration was recorded directly from the frozen run rather than reconstructed from memory. Experiments used Windows 11 (build 26200), an Intel Core Ultra 7 270K Plus processor (24 cores), 63.4 GiB of system random-access memory (RAM), and an NVIDIA GeForce RTX 5090 D v2. The formal training device was CPU; the GPU was used only for separately labeled batch-1 latency profiling. The software stack was Python 3.10.12, PyTorch 2.11.0+cu130 with Compute Unified Device Architecture (CUDA) build 13.0, NumPy 2.2.6, pandas 2.3.3, and SciPy 1.15.3. These versions are also written to the run manifest. Table 3 fixes the environment scale and run counts used in all comparisons; these are protocol constants rather than outcomes selected during analysis.

The environment follows the observation and file-level protocol in Section 2. Exploration is disabled during evaluation, and each learned policy acts greedily. For each evaluation-only physical condition and jammer mode, the formal endpoint uses a scan-stratified set of 20 fixed trajectories: each of the ten scan IDs contributes exactly two replicates. Every method receives the same scan ID, start index, jammer realization, and trajectory seed. The 20 cm/10 dBm scan IDs 8–9 are explicitly a pre-inspected pilot. The 40 cm/10 dBm and 20 cm/5 dBm configurations are outcome-uninspected evaluation-only transfer conditions. They were declared by file names before any policy outcome was computed; format and finite-value auditing did not expose policy performance. Code, thresholds, hyperparameters, and the analysis plan were frozen before transfer outcomes were inspected. The dated protocol amendment records the scan-stratified design and the stronger sweep-aware reference, which were both specified before transfer outcomes were inspected.

The shared optimization hyperparameters are summarized in Table 4. HNP-DQN, the capacity-matched MLP-DDQN, and learned ablations use the same optimizer, replay, interaction budget, exploration schedule, target update, training seeds, and evaluation trajectories wherever the mechanism applies. HNP-DQN uses a second-order element-wise expansion; all learned agents use the same Adam weight decay ($1\times 10^{-4}$). The frozen protocol did not permit configuration changes selected on the basis of transfer performance.

### 4.2. Endpoints and Statistical Analysis

For each independently trained seed, the primary endpoint is the mean 100-step evaluation return across the fixed trajectories. Collision rate and switch rate are supporting behavioral endpoints. Training time, batch-1 CPU/GPU inference latency, trainable parameters, serialized model size, and persistent model-tensor footprint are implementation endpoints. For each jammer mode, the protocol declares a primary rule comparator: the schedule-aware rule with a declared sweep specification and internally tracked progression is used under deterministic sweeping, whereas the threshold/hysteresis rule is used under random jamming. These comparator choices were fixed before the two transfer outcomes were inspected; the within-configuration pilot had been inspected previously and is labeled accordingly. The HNP-DQN versus capacity-matched MLP-DDQN comparison is primary under both modes. The clairvoyant oracle and jammer-aware greedy controller are privileged descriptive references outside the primary multiplicity family; other method comparisons and component ablations are secondary or exploratory.

Every seed is reported. The independent experimental unit is the trained seed; it is not an episode or time step. For each paired comparison, the analysis reports the paired mean difference, its 95% CI, a paired standardized effect size, and a two-sided exact sign-flip/permutation result using all completed seed pairs. Holm correction is applied across the declared comparison family. The analysis used 10 paired training seeds and 20 fixed evaluation trajectories per seed, physical condition, and jammer mode. Confidence intervals are two-sided 95% Student-*t* intervals for the paired seed differences, effect sizes are Cohen’s $d_{z}$, and *p*-values use two-sided exact sign-flip enumeration. Holm correction is applied to one predeclared primary family containing 12 return comparisons. A separate 24-comparison exploratory Holm family is used for the four component variants.

### 4.3. Comparative Experiments

The primary learned-model comparison is between HNP-DQN and the capacity-matched MLP-DDQN. Component deletions and the γ=0 HNP-DQN diagnostic are secondary analyses. All learned methods receive the same 48-dimensional observation and share the declared training and evaluation protocol.

The following non-learning policies test whether a deep value function is needed:

1. **Switching-aware threshold/hysteresis.** The policy retains the current channel while its development-defined score satisfies the threshold and otherwise changes to the best currently admissible channel. The threshold is the predeclared median (q=0.5) of the quality scores pooled from the training and validation files; it is not selected by a post hoc grid search. The frozen threshold is 4.4879. At reset, or whenever the previous channel falls below this value, the policy selects the maximum current quality; exact ties choose the lowest action index.
2. **Maximum measured quality/minimum interference.** At every step, the rule selects the channel ranked best by the development-verified direction of the dataset-provided snr feature. Specifically, it chooses the smallest current raw snr_mean, equivalently the largest −snr_mean; exact ties choose the lowest action index.
3. **Stay on current channel.** After the declared initialization rule, the policy retains the same channel for the episode. It starts at action 0 (5180 MHz) and never changes action.
4. **Random Selection.** The policy samples uniformly from the eight actions.
5. **Schedule-aware sweeping rule.** This deterministic rule is evaluated only under the declared sweep. It uses the declared initial phase, one-channel-per-step direction, and an internal step counter to track the deterministic progression; it neither reads the RF observation nor the simulator’s runtime jammer label. It retains a safe channel and, immediately before that channel is swept, changes to the channel that was swept in the preceding step. Unlike HNP-DQN and the ordinary observation-based baselines, this rule receives the additional schedule information described above. These schedule variables and the corresponding step index are not included in the 48-dimensional agent observation. Its comparison with HNP-DQN is therefore an information-asymmetric operational comparison that provides a reference for directly exploiting a known deterministic schedule structure rather than a matched-information architectural comparison. It is not assumed to be deployable when the phase or schedule is unknown.
6. **Jammer-aware greedy reference.** This privileged controller may read the current simulated jammer label. It retains its previous action if safe and otherwise selects the highest measured-quality non-jammed channel. Because it sees only the current label and optimizes greedily, it is not described as an upper bound.
7. **Clairvoyant dynamic-programming (DP) upper bound.** This non-deployable reference receives the complete realized jammer-label sequence and solves the finite-horizon action sequence that maximizes the stated reward, including switching cost. It supplies a mathematical upper bound for each fixed trajectory rather than an ordinary policy comparator.

All ordinary observation-based deterministic policies use only the 48-dimensional observation. The threshold and any other tunable rule are fixed using training and validation files only. The schedule-aware and privileged references use only the additional information explicitly stated above. Every method is evaluated on the same fixed, scan-stratified trajectories.

Table 5 distinguishes the predeclared comparators by their information access. The schedule-aware rule uses the known deterministic sweep schedule and is therefore not an ordinary observation-only heuristic. It matched the trajectory-wise DP upper bound in all three sweeping settings (return 98.60, collision rate 0%, and switch rate 14%). By contrast, HNP-DQN returned 96.69±0.83, 95.09±1.44, and 96.63±0.87 in the pilot, distance-transfer, and power-transfer sweeping settings, respectively, with higher switching rates of 31.03±8.82%, 38.37±10.99%, and 29.92±8.93%. Under random jamming, the return differences between HNP-DQN and the threshold rule (HNP-DQN minus threshold) were 0.5020, −1.1745, and 0.5885 across the same three settings; none survived family-wise Holm correction. The corresponding collision-rate differences were +0.075, +0.075, and −0.095 percentage points, and the switch-rate differences were −6.520, +10.280, and −3.970 percentage points.

As summarized in Table 6, statistical inference is based exclusively on the displayed seed-level values with one independently trained seed treated as the inferential unit. A nonsignificant result is described as inconclusive rather than evidence of equivalence. After Holm correction, three of the 12 primary comparisons favored the schedule-aware rule, and the remaining nine were inconclusive.

Relative to the schedule-aware rule under deterministic sweeping, HNP-DQN had lower return in the pilot (Δ=−1.9100, 95% CI [−2.5012,−1.3188], $d_{z}=-2.311$), distance-transfer (Δ=−3.5125, [−4.5424,−2.4826], $d_{z}=-2.440$), and power-transfer (Δ=−1.9700, [−2.5905,−1.3495], $d_{z}=-2.271$) settings; all three comparisons had $p_{\mathrm{Holm}}=0.023438$. These three results are conditional on the schedule-aware rule’s use of the declared initial sweep phase and one-channel-per-step direction together with an internal step counter that tracks deterministic progression. The schedule variables and corresponding step index are absent from HNP-DQN’s observation. The comparison therefore shows the operational advantage attainable when the known sweep specification is directly exploited; it should not be interpreted as a matched-information ranking between a deterministic rule and the HNP-DQN architecture.

Against the threshold rule under random jamming, the differences were 0.5020 ([−0.0387,1.0427], $p_{\mathrm{Holm}}=0.500000$), −1.1745 ([−2.1081,−0.2409], $p_{\mathrm{Holm}}=0.210938$), and 0.5885 ([−0.1376,1.3146], $p_{\mathrm{Holm}}=0.574219$). Although the unadjusted distance-transfer interval excluded zero, that comparison was not significant after correction and is not treated as confirmatory evidence. All six comparisons with the capacity-matched MLP were likewise inconclusive: their Holm-adjusted *p*-values ranged from 0.500000 to 0.789063, and every confidence interval included zero.

The primary results therefore support a conditional interpretation: explicit deterministic schedule knowledge provides clear decision value when available, whereas under observation-matched random interference and in the approximately parameter-matched neural comparison, the present experiment did not resolve a reliable return difference.

**Figure 7:**
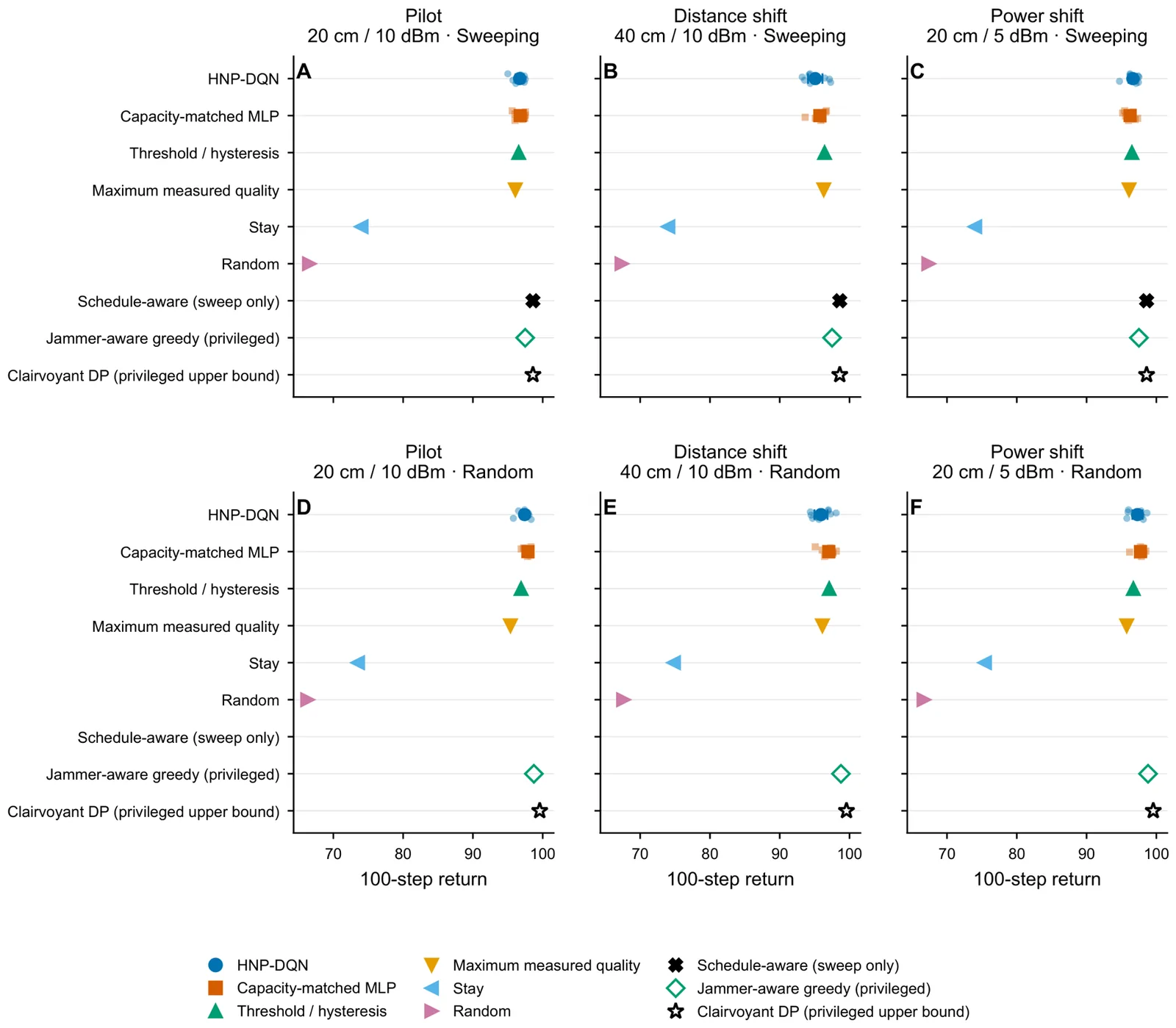
Main performance under the frozen scan-stratified protocol. Columns show the pre-inspected 20 cm/10 dBm pilot and the outcome-uninspected 40 cm/10 dBm distance and 20 cm/5 dBm power transfers; rows show sweeping and random jammer modes. For HNP-DQN and the capacity-matched MLP-DDQN, faint markers show the 10 independently trained seed estimates after averaging 20 fixed trajectories per seed, while the larger markers and error bars show the seed means and their two-sided 95% Student-*t* confidence intervals. Deterministic references are shown as fixed-trajectory means without seed confidence intervals. The schedule-aware rule is evaluated only under sweeping; hollow markers identify privileged references. Pilot scans 8 and 9 occur 10 times each, whereas transfer scans 0–9 occur twice each. Panels (**A**–**C**) show the pilot, distance-transfer, and power-transfer results under sweeping jamming, respectively; panels (**D**–**F**) show the corresponding results under random jamming.

**Figure 8:**
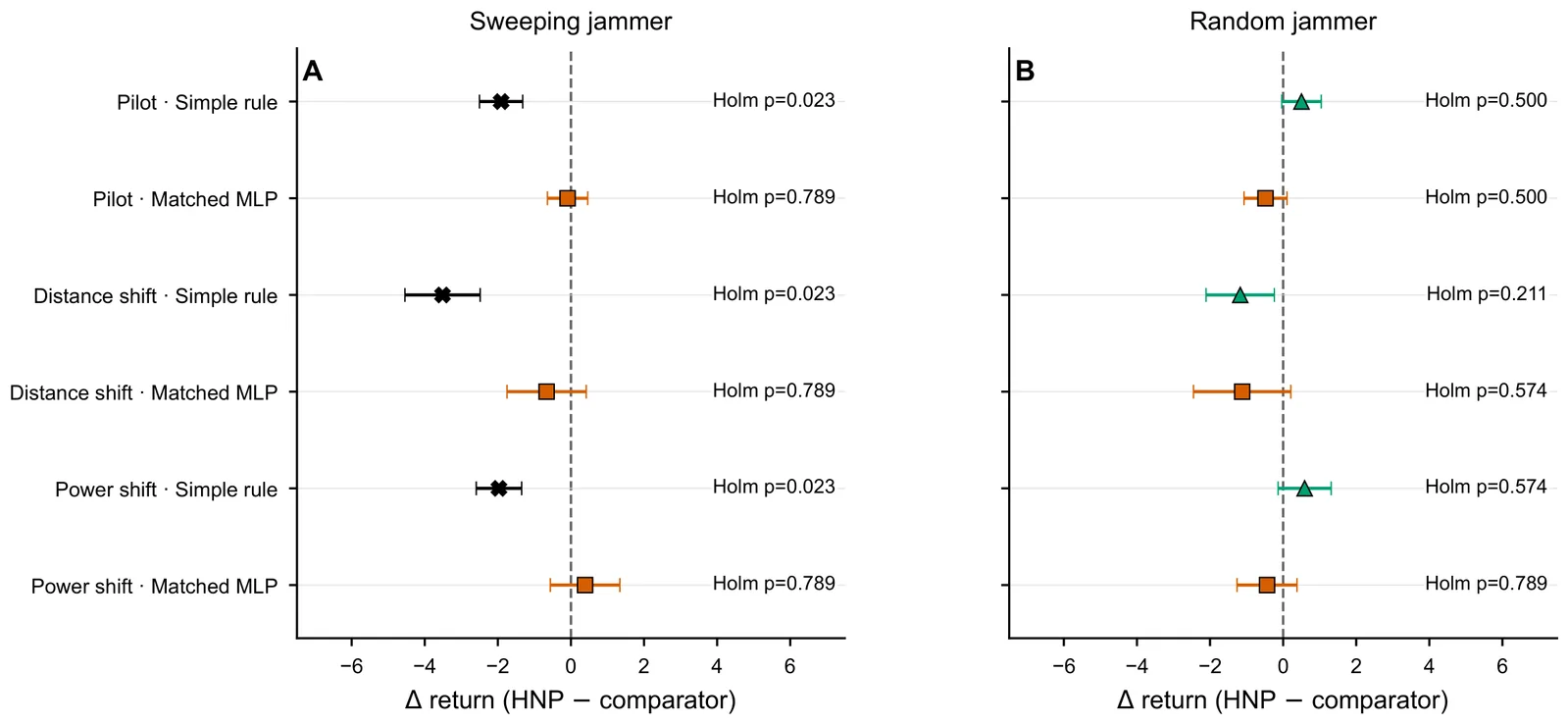
Predeclared primary paired comparisons. Points show the mean difference in 100-step return, HNP-DQN minus comparator, across 10 paired training seeds; horizontal bars are two-sided 95% Student-*t* confidence intervals. Under sweeping, the predeclared rule comparator is the schedule-aware rule with a declared sweep specification and internally tracked step progression; under random jamming, it is the threshold/hysteresis rule. The capacity-matched MLP-DDQN is compared under both modes. The dashed line denotes no difference, and numeric labels are Holm-adjusted *p*-values for the single 12-comparison primary family. The pilot is distinguished from the two cross-configuration evaluations. Panel (**A**) shows the sweeping-jammer comparisons, and panel (**B**) shows the random-jammer comparisons. Black X markers, green triangles, and orange squares denote the schedule-aware rule, the threshold/hysteresis rule, and the capacity-matched MLP-DDQN, respectively.

Figure 7 shows method-wise returns across the six physical-condition and jammer-mode settings, whereas Figure 8 isolates the 12 primary HNP-minus-comparator paired differences with their 95% confidence intervals. Together, the figures separate the information-asymmetric schedule-aware comparisons from the observation-matched random and capacity-matched comparisons; inconclusive intervals are not interpreted as evidence of equivalence.

Cross-model TD-loss magnitude is not used as evidence that the polynomial mapping or LayerNorm is more stable. Different architectures and bootstrap targets can produce different loss scales. TD loss remains an internal optimization diagnostic only. We report seed-level return dispersion and failed-run counts descriptively; however, we did not predeclare a separate convergence or area-under-the-learning-curve endpoint. We therefore make no architecture-level stability claim from the present experiment. All 10 canonical training seeds were present for every learned method and setting, and artifact validation found no failed or incomplete run in the canonical schedules. Across the six primary settings, the between-seed standard deviation of HNP-DQN return ranged from 0.7559 to 1.4397, whereas that of the capacity-matched MLP ranged from 0.4646 to 0.8972. These values describe endpoint variability across the frozen canonical runs and are not used to support a convergence, area-under-learning-curve, or cross-model TD-loss claim.

### 4.4. Model Capacity and Computational Cost

The cost analysis reports trainable parameters, the persistent tensor footprint of parameters and buffers, serialized state/checkpoint size, training time, and batch-1 CPU and GPU inference latency under a stated warm-up and repetition protocol. The persistent tensor footprint excludes framework, allocator, and activation overhead and is not reported as peak runtime memory. We do not report unverified multiply–accumulate (MAC) or floating-point operation (FLOP) estimates.

All six capacity-matched HNP-DQN versus MLP-DDQN comparisons were inconclusive after Holm correction with every confidence interval including zero and adjusted *p*-values ranging from 0.500000 to 0.789063. The close parameter-count match reduces a simple model-size explanation, while the present experiment does not statistically resolve a reliable held-out return difference between HNP-DQN and this approximately parameter-matched conventional control. The matched MLP used 150 fewer parameters (−0.46% relative to HNP-DQN), while its persistent parameter/buffer footprint was slightly larger (130.07 versus 129.80 KiB). HNP-DQN had larger serialized state/checkpoint sizes, higher median batch-1 latencies on both the CPU and GPU, and a longer median training time in this engineering profile. Together, these measurements define a transparent implementation reference for evaluating the structured representation in richer nonlinear or temporally coupled settings, while the current held-out comparison remains statistically unresolved.

Latency values in Table 7 are medians [25th, 75th percentiles] across 20 checkpoint-level medians per model, using 200 warm-up and 1000 timed repetitions with one CPU thread; training times are medians [25th, 75th percentiles] across three engineering timing seeds per model. A 5 ms GPU inter-iteration pacing sleep was applied only after each forward pass had been synchronized and its latency sample recorded; the sleep lies outside the timed region and is excluded. These timing summaries are engineering measurements; they are not inferential endpoints. Persistent tensor size includes parameters and registered buffers only; it excludes activations, optimizer state, framework/runtime objects, temporary workspaces, and allocator overhead and is not peak runtime memory.

### 4.5. Ablation Study

The ablation study uses the same file partition, training-seed schedule, and fixed evaluation trajectories as the primary comparison. It considers the full HNP-DQN, removal of the element-wise polynomial branch, removal of LayerNorm, removal of the dueling head, the capacity-matched non-polynomial MLP-DDQN, and an otherwise matched HNP-DQN with γ=0. The approximately parameter-matched conventional control provides a complementary parameter-count check because a narrower no-polynomial ablation confounds representation form with parameter count. The γ=0 variant tests whether long-horizon bootstrapping adds value beyond myopic state-conditioned selection. All variants retain the 48-dimensional observation, DDQN target construction, replay settings, optimizer, and stopping rule. Comparisons are paired at the training-seed level. Ablations form a separate exploratory Holm family and are not mixed with the primary comparison family.

As summarized in Table 8, all 10 canonical training seeds and the frozen code, checkpoint, normalizer, and evaluation-schedule hashes passed the artifact gate for each ablation run. The 24-comparison exploratory ablation family yielded one family-wise significant result and 23 inconclusive comparisons. The sole family-wise significant result occurred in the random-pilot setting, where the γ=0 variant outperformed HNP-DQN: $\Delta_{\mathrm{HNP}-\gamma =0}=-1.3275$ (95% CI [−1.9024,−0.7526], $d_{z}=-1.652$, $p_{\mathrm{Holm}}=0.046875$). The γ=0 variant achieved 98.7295±0.1848 compared with 97.4020±0.7559 for HNP-DQN; HNP-DQN also had a collision rate higher by 0.070 percentage points and a switch rate higher by 11.875 percentage points in this setting. Apparent HNP-DQN advantages over γ=0 in the distance-transfer settings were not family-wise significant, including deterministic sweeping (Δ=5.5815, $p_{\mathrm{Holm}}=0.890625$) and random jamming (Δ=6.5890, $p_{\mathrm{Holm}}=0.644531$); the γ=0 ablation also showed substantial seed variability in those transfer settings. Removing the polynomial branch produced confidence intervals containing zero in all six settings, while the corresponding No-LayerNorm and No-Dueling component effects were likewise not statistically resolved after Holm correction. The six matched-MLP rows belong to the primary family and were all inconclusive. These data neither demonstrate the necessity of any individual component nor establish equivalence. Training return and TD loss are retained only as optimization diagnostics and are not used to rank architectural variants.

**Figure 9:**
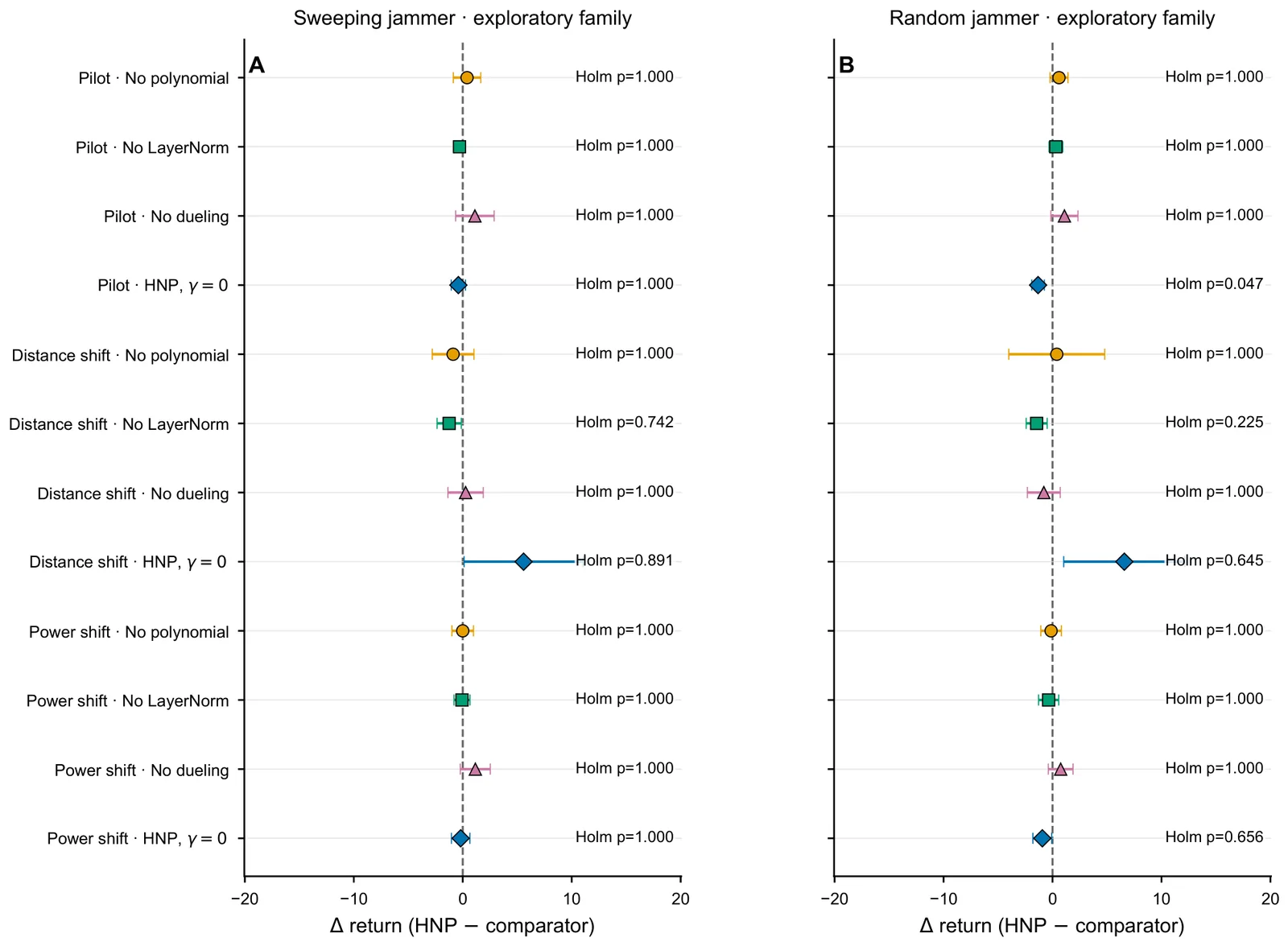
Exploratory paired ablation comparisons. Points show the mean difference in 100-step return, full HNP-DQN minus variant, across 10 paired training seeds; horizontal bars are two-sided 95% Student-*t* confidence intervals. The dashed line denotes no difference. Numeric labels are Holm-adjusted *p*-values for the separate 24-comparison exploratory family spanning four variants, three physical conditions, and two jammer modes. Nonsignificant results are interpreted as inconclusive rather than as evidence of equivalence. Panel (**A**) shows the sweeping-jammer ablations, and panel (**B**) shows the random-jammer ablations. Circles, squares, triangles, and diamonds denote the No-Polynomial, No-LayerNorm, No-Dueling, and γ=0 variants, respectively.

Figure 9 visualizes these paired return differences and their uncertainty; intervals that cross zero remain inconclusive and are not interpreted as equivalence.

### 4.6. Post Hoc Analysis of Expanded-Feature Weights

The deterministic polynomial mapping forms $\left[h,h^{⊙2}\right]$ before the learned projection. We audited the frozen projection.0.weight tensor, whose verified shape was 128×128, in 20 HNP-DQN checkpoints (10 seeds for each jammer mode). Columns 0–63 were assigned to the normalized linear branch and columns 64–127 to the normalized squared branch. Across the 20 seed–mode checkpoints, the mean absolute outgoing weight was 0.021736±0.000980 for the linear branch and 0.029715±0.001602 for the squared branch; the paired descriptive squared-minus-linear difference was 0.007979±0.001602. The corresponding Frobenius norms were 3.3549±0.1013 and 4.4298±0.1287. By jammer mode, the linear/squared mean absolute weights were 0.021364±0.000931/0.029310±0.001849 under sweeping and 0.022109±0.000924/0.030121±0.001278 under random jamming.

These are descriptive raw-weight magnitudes only; no inferential test or association with policy performance was performed. The magnitudes depend on scale and reparameterization. Moreover, the branch weights are coupled through the learned LayerNorm and upstream activation scales, and the analysis ignores activation frequency, sign cancellation, and downstream nonlinear effects. They therefore do not constitute evidence of causal feature importance, branch contribution, robustness, or interpretability [37,38]. In particular, this descriptive audit is not used as evidence of a causal or performance contribution from the polynomial branch. Action-specific claims would require evidence from controlled masking or perturbation on fixed trajectories and remain outside the scope of this paper.

## 5. Conclusions

### 5.1. Summary

This paper develops and evaluates HNP-DQN as a structured value-function approximator for an eight-channel, measurement-driven spectrum-selection testbed. The architecture combines a learned latent representation with an element-wise second-order expansion, LayerNorm, and a dueling DDQN backbone. The accompanying protocol makes the previous-channel switching state explicit and evaluates the model through file-level separation, common fixed trajectories, independently trained seeds, an approximately parameter-matched neural control, strong deterministic policies, and multiplicity-corrected paired inference.

The evaluation reveals an information-dependent result. Under deterministic sweeping, the schedule-aware rule receives the declared initial sweep phase and one-channel-per-step direction and uses an internal step counter to track the deterministic progression; these schedule variables and the corresponding step index are not included in HNP-DQN’s 48-dimensional observation. The rule matched the trajectory-wise dynamic-programming upper bound and outperformed HNP-DQN in all three settings with differences calculated as HNP-DQN minus the rule of −1.9100, −3.5125, and −1.9700 (all $p_{\mathrm{Holm}}=0.023438$). This information-asymmetric operational comparison shows the advantage attainable when the known sweep specification is directly exploited rather than a matched-information architectural ranking. Under random jamming, the three comparisons with the observation-based threshold rule were inconclusive after Holm correction. Likewise, all six comparisons with the capacity-matched MLP were inconclusive. Because the models also differ in hidden widths and normalization paths, this approximately parameter-matched comparison does not isolate the squared-feature branch. Instead, it provides a rigorous architectural reference, although the held-out return difference remains unresolved. The exploratory ablations provide one setting-specific result favoring γ=0 in the random pilot, while the remaining 23 comparisons are inconclusive.

Taken together, these findings define a controlled model-selection reference within the declared measurement-replay testbed. Reliable deterministic interference information should be exploited directly when it is available; when only measurement observations are available, new representations should be tested against strong observation-based rules and approximately parameter-matched neural controls. The present results do not show that a DQN is required merely because a switching cost introduces temporal coupling. Rather, they identify more strongly action-dependent settings, as well as partially observed, reactive, and transfer settings, in which the decision value of the structured representation and long-horizon learning can be tested more decisively. The contribution is therefore not a universal superiority claim; instead, it is an HNP-DQN architecture and a reproducible framework for evaluating whether additional information or learning complexity is justified.

### 5.2. Limitations and Future Work

The protocol provides a raw-file-disjoint development split and two declared cross-configuration evaluations, while independent acquisition-session and new-site confirmation remain outside the present dataset. At 20 cm/10 dBm, scans 0–6 are used for training and scan 7 is used for validation; scans 8–9 are labeled a pre-inspected pilot because they were used in an earlier engineering-rule diagnostic. The 40 cm/10 dBm and 20 cm/5 dBm file sets are evaluation-only transfer conditions. They were declared by file names before any policy outcome was computed and are therefore outcome-uninspected, although the files could be loaded beforehand for format and finite-value auditing. This paper has no acquisition-session-disjoint, new-site, or independent external confirmation set, so robustness beyond these two controlled shifts is not claimed.

The measurements form a controlled communication-system RF testbed rather than an end-to-end radar experiment. The dataset-provided snr field is not treated as a calibrated physical SNR or SINR, and this paper does not include radar waveforms, target echoes, detection or false-alarm probability, tracking, or hardware-in-the-loop operation. Consequently, “radar-oriented” describes the intended decision-layer use case rather than hardware validation.

The measured RF sequence is exogenous: selecting a channel changes only the stored previous action and hence the later switching penalty. This controlled setting does not test an action-dependent or reactive jammer. Reactive, deceptive, learning-based, multi-jammer, partially observed, and delayed environments remain open tests of whether sequential value learning provides an advantage over contextual and deterministic policies [39]. Moreover, each fixed-jammer source file lacks verified timestamps shared with the other jammer-labeled files. The sweeping and random trajectories are therefore synthetic replay sequences constructed from ordinal indices; they are not continuous time-synchronized measurements of a moving jammer. This limits the evidence to policy behavior in the declared replay simulator.

The normalized coefficient $C_{\mathrm{switch}}=0.1$ is fixed rather than calibrated to retuning latency, energy, synchronization delay, or sensing loss. The current comparisons therefore characterize one declared operating preference; broader sensitivity analysis and hardware-based calibration are needed before attaching physical meaning to this penalty. Likewise, the reported parameter, timing, and memory measurements describe only the frozen software/hardware stack and do not establish embedded-device suitability.

Finally, seed-level pairing quantifies algorithmic variability across the available scan files but cannot estimate population variability across independent measurement campaigns. Weight magnitudes and TD loss are retained only as descriptive training diagnostics. Causal or action-specific interpretability would require intervention on fixed trajectories, such as masking or perturbation, and is not claimed here.

These extensions would provide direct tests of whether the information- and temporal-structure-dependent findings identified here transfer to more interactive sensing and spectrum-control environments.

## Figures and Tables

**Table 1:** Evolution of value-based reinforcement learning algorithms, including temporal-difference (TD) learning, deep Q-network (DQN) variants, and prioritized experience replay (PER).

Algorithm	Year	Core Contribution
TD learning	1988	Bootstrapped value estimation
Q-learning	1992	Off-policy Bellman update
DQN	2015	Deep neural Q-approximation
Double DQN	2016	Reduces overestimation bias
Dueling DQN	2016	Value–advantage stream decomposition
PER	2016	Priority-weighted experience replay
Rainbow	2018	Integrated multi-improvement agent

**Table 2:** Frozen file-level development and evaluation protocol. Counts are 32-sample, stride-1 windows generated separately within each raw source file.

Distance/Power	Scan IDs	Role	Window Count
20 cm/10 dBm	0–6	Training	36,790
20 cm/10 dBm	7	Validation	5256
20 cm/10 dBm	8–9	Pre-inspected pilot	10,511
40 cm/10 dBm	0–9	Evaluation-only distance transfer	52,613
20 cm/5 dBm	0–9	Evaluation-only power transfer	52,592

**Table 3:** Parameters of the environment.

Parameter	Value
Channels/frequency range	8/5180–5320 MHz
Channel spacing/window	20 MHz/32 samples, stride 1
Development configuration	20 cm/10 dBm
Evaluation-only transfers	40 cm/10 dBm; 20 cm/5 dBm
Observation dimension	48 (40 RF + 8 previous-channel)
Channel switching cost	0.1 (fixed normalized value)
Maximum steps per episode	100
Training episodes	200
Fixed evaluation seeds	1001–1020
Completed independent training seeds	10 per learned method and jammer mode

**Table 4:** Hyperparameters of the deep Q-network (DQN)-family agents, including the capacity-matched multilayer perceptron (MLP).

Parameter	Value
Learning rate α	0.001
Optimizer/TD objective	Adam/mean-squared TD error
Discount factor γ	0.95
Soft update rate τ	0.005
Replay buffer *N*	100,000
Batch size *B*	64
Learning start/update frequency	64 steps/every step
Gradient clipping threshold	0.7
Exploration schedule	max(0.01,1/(1+e/10)) per episode
HNP-DQN feature widths	[128, 64]
HNP-DQN expansion order	2 (element-wise, no cross terms)
Adam weight decay	$1\times 10^{-4}$
Capacity-matched MLP widths	[174, 128]

**Table 5:** Performance of the core methods under the frozen file-level protocol. Each cell reports the 100-step return on the first line and the collision and switch rates (C/S; %) on the second line. Learned-model entries are the mean ± sample standard deviation (SD) across 10 independently trained seeds, which are each averaged over the same 20 fixed trajectories. Fixed-rule entries are descriptive means over those 20 trajectories and therefore have no training-seed SD. The predeclared rule is schedule-aware (S) under sweeping and threshold/hysteresis (T) under random jamming. Rule = dynamic programming (DP) denotes equality of the displayed return/collision/switch summaries with the clairvoyant DP upper bound.

Setting	Mode	Learned: Mean ± SD (n=10)		Fixed-Rule Description	
		HNP-DQN Return; C/S	Matched MLP Return; C/S	Primary Rule Return; C/S	Rule = DP?
Pilot 20 cm/10 dBm	Sweep	96.69 ± 0.83 0.11 ± 0.25/31.03 ± 8.82	96.78 ± 0.71 0.09 ± 0.12/30.56 ± 7.09	S: 98.60 0.00/14.00	Yes
	Random	97.40 ± 0.76 0.08 ± 0.24/24.48 ± 7.13	97.88 ± 0.46 0.11 ± 0.18/19.03 ± 2.54	T: 96.90 0.00/31.00	n/a
Distance transfer 40 cm/10 dBm	Sweep	95.09 ± 1.44 0.56 ± 0.55/38.37 ± 10.99	95.75 ± 0.86 0.50 ± 0.27/32.81 ± 5.36	S: 98.60 0.00/14.00	Yes
	Random	95.91 ± 1.31 0.08 ± 0.07/39.43 ± 12.13	97.03 ± 0.90 0.06 ± 0.04/28.59 ± 8.81	T: 97.09 0.00/29.15	n/a
Power transfer 20 cm/5 dBm	Sweep	96.63 ± 0.87 0.20 ± 0.24/29.92 ± 8.93	96.24 ± 0.71 0.22 ± 0.17/33.42 ± 7.34	S: 98.60 0.00/14.00	Yes
	Random	97.29 ± 1.02 0.11 ± 0.30/25.03 ± 8.87	97.73 ± 0.64 0.03 ± 0.08/22.22 ± 6.89	T: 96.70 0.20/29.00	n/a

*Note*: n/a means not applicable; Rule = DP is assessed only for the schedule-aware sweeping rule.

**Table 6:** Primary paired seed-level comparisons. Paired differences are calculated as HNP-DQN minus the listed comparator; HNP in the compact row labels denotes HNP-DQN. Adjusted *p*-values use Holm correction across the 12 predeclared physical-condition, jammer-mode, and comparator rows.

Condition/Comparison	Paired Difference (95% Confidence Interval)	Effect Size	Holm-Adjusted Inference
*Sweeping: schedule-aware comparator with declared schedule and tracked progression*			
20 cm/10 dBm pilot: HNP vs. schedule-aware	−1.9100 [−2.5012, −1.3188]	$d_{z}=-2.311$	HNP lower; $p_{\mathrm{Holm}}=0.023438$
20 cm/10 dBm pilot: HNP vs. matched MLP	−0.0910 [−0.6423, 0.4603]	$d_{z}=-0.118$	Inconclusive; $p_{\mathrm{Holm}}=0.789063$
40 cm/10 dBm transfer: HNP vs. schedule-aware	−3.5125 [−4.5424, −2.4826]	$d_{z}=-2.440$	HNP lower; $p_{\mathrm{Holm}}=0.023438$
40 cm/10 dBm transfer: HNP vs. matched MLP	−0.6645 [−1.7482, 0.4192]	$d_{z}=-0.439$	Inconclusive; $p_{\mathrm{Holm}}=0.789063$
20 cm/5 dBm transfer: HNP vs. schedule-aware	−1.9700 [−2.5905, −1.3495]	$d_{z}=-2.271$	HNP lower; $p_{\mathrm{Holm}}=0.023438$
20 cm/5 dBm transfer: HNP vs. matched MLP	0.3875 [−0.5635, 1.3385]	$d_{z}=0.291$	Inconclusive; $p_{\mathrm{Holm}}=0.789063$
*Random: predeclared observation-based comparator = threshold/hysteresis*			
20 cm/10 dBm pilot: HNP vs. threshold	0.5020 [−0.0387, 1.0427]	$d_{z}=0.664$	Inconclusive; $p_{\mathrm{Holm}}=0.500000$
20 cm/10 dBm pilot: HNP vs. matched MLP	−0.4825 [−1.0700, 0.1050]	$d_{z}=-0.587$	Inconclusive; $p_{\mathrm{Holm}}=0.500000$
40 cm/10 dBm transfer: HNP vs. threshold	−1.1745 [−2.1081, −0.2409]	$d_{z}=-0.900$	Inconclusive after Holm; $p_{\mathrm{Holm}}=0.210938$
40 cm/10 dBm transfer: HNP vs. matched MLP	−1.1210 [−2.4527, 0.2107]	$d_{z}=-0.602$	Inconclusive; $p_{\mathrm{Holm}}=0.574219$
20 cm/5 dBm transfer: HNP vs. threshold	0.5885 [−0.1376, 1.3146]	$d_{z}=0.580$	Inconclusive; $p_{\mathrm{Holm}}=0.574219$
20 cm/5 dBm transfer: HNP vs. matched MLP	−0.4400 [−1.2598, 0.3798]	$d_{z}=-0.384$	Inconclusive; $p_{\mathrm{Holm}}=0.789063$

**Table 7:** Model capacity and implementation cost under a common 48-dimensional input. Central processing unit (CPU) and graphics processing unit (GPU) latencies are reported in milliseconds. Bracketed values are the 25th and 75th percentiles.

Method	Parameters/Persistent Tensors	Serialized Size	Latency/Training Time
HNP-DQN	32,841 trainable parameters 129.80 KiB persistent tensors	state (KiB) 136.91 [136.91, 136.91] checkpoint (KiB) 545.14 [545.14, 545.14]	CPU (ms) 0.118 [0.113, 0.122] GPU (ms) 1.403 [1.374, 1.426] training (s) 79.554 [77.941, 80.998]
Capacity-matched MLP-DDQN	32,691 trainable parameters 130.07 KiB persistent tensors	state (KiB) 136.04 [136.04, 136.04] checkpoint (KiB) 538.20 [538.20, 538.20]	CPU (ms) 0.084 [0.073, 0.087] GPU (ms) 1.159 [1.135, 1.184] training (s) 47.529 [47.504, 52.205]

**Table 8:** Seed-level architecture comparisons. Each entry reports the paired return difference (full HNP-DQN minus variant) with a 95% Student-*t* confidence interval across 10 paired training seeds. The six matched-MLP entries belong to the predeclared 12-comparison primary Holm family; the other 24 entries belong to the separate exploratory ablation Holm family. Collision and switching outcomes are descriptive supporting endpoints and are not added to either return family.

Variant/Jammer	20 cm/10 dBm Pilot	40 cm/10 dBm Transfer	20 cm/5 dBm Transfer
No polynomial/sweeping	0.393 [−0.866, 1.653]	−0.888 [−2.801, 1.025]	−0.005 [−0.992, 0.983]
No polynomial/random	0.595 [−0.231, 1.421]	0.389 [−4.011, 4.789]	−0.132 [−1.071, 0.807]
No LayerNorm/sweeping	−0.297 [−0.843, 0.250]	−1.246 [−2.356, −0.137]	−0.072 [−0.802, 0.658]
No LayerNorm/random	0.308 [−0.325, 0.940]	−1.456 [−2.421, −0.491]	−0.356 [−1.282, 0.570]
No dueling/sweeping	1.113 [−0.657, 2.883]	0.260 [−1.362, 1.883]	1.153 [−0.223, 2.530]
No dueling/random	1.091 [−0.154, 2.336]	−0.799 [−2.310, 0.713]	0.747 [−0.390, 1.885]
Matched MLP/sweeping	−0.091 [−0.642, 0.460]	−0.664 [−1.748, 0.419]	0.387 [−0.563, 1.338]
Matched MLP/random	−0.482 [−1.070, 0.105]	−1.121 [−2.453, 0.211]	−0.440 [−1.260, 0.380]
γ=0 HNP/sweeping	−0.404 [−1.056, 0.248]	5.582 [0.120, 11.043]	−0.193 [−1.039, 0.653]
γ=0 HNP/random	−1.327 [−1.902, −0.753]	6.589 [1.029, 12.149]	−0.937 [−1.799, −0.076]

## Data Availability

The source code developed for this paper is publicly available at https://github.com/TheRyans520/HNPDQN (accessed on 27 August 2026). The RF Jamming dataset used in this paper is publicly available and documented by Ali et al. in “RF Jamming Dataset: A Wireless Spectral Scan Approach for Malicious Interference Detection”.

## Abbreviations

The following abbreviations are used in this manuscript:

BN	Batch Normalization
CEW	Cognitive Electronic Warfare
CI	Confidence Interval
CPU	Central Processing Unit
CSV	Comma-Separated Values
CUDA	Compute Unified Device Architecture
DDQN	Double Deep Q-Network
DP	Dynamic Programming
DQN	Deep Q-Network
DRL	Deep Reinforcement Learning
EP	Electronic Protection
FLOP	Floating-Point Operation
GPU	Graphics Processing Unit
HNP-DQN	Hybrid Neural-Polynomial Deep Q-Network
HT	High Throughput
LayerNorm	Layer Normalization
MAC	Multiply–Accumulate Operation
MDP	Markov Decision Process
MLP	Multilayer Perceptron
MSE	Mean Squared Error
PER	Prioritized Experience Replay
RAM	Random-Access Memory
ReLU	Rectified Linear Unit
RF	Radio Frequency
RFI	Radio Frequency Interference
RL	Reinforcement Learning
RSSI	Received Signal Strength Indicator
SD	Standard Deviation
SINR	Signal-to-Interference-plus-Noise Ratio
SNR	Signal-to-Noise Ratio
TD	Temporal Difference
VHT	Very High Throughput
